# Why has farming in Europe changed? A farmers’ perspective on the development since the 1960s

**DOI:** 10.1007/s10113-023-02150-y

**Published:** 2023-11-11

**Authors:** Franziska Mohr, Vasco Diogo, Julian Helfenstein, Niels Debonne, Thymios Dimopoulos, Wenche Dramstad, Maria García-Martín, Józef Hernik, Felix Herzog, Thanasis Kizos, Angela Lausch, Livia Lehmann, Christian Levers, Robert Pazur, Virginia Ruiz-Aragón, Rebecca Swart, Claudine Thenail, Hege Ulfeng, Peter H. Verburg, Tim Williams, Anita Zarina, Matthias Bürgi

**Affiliations:** 1 Land Change Science Research Unit, Swiss Federal Research Institute WSL, Zürcherstrasse 111, Birmensdorf, Switzerland; 2 Institute of Geography, University of Bern, Bern, Switzerland; 3 Agroecology and Environment, Agroscope, Zurich, Switzerland; 4 Soil Geography and Landscape Group, Wageningen University, Wageningen, The Netherlands; 5 Environmental Geography Group, Institute for Environmental Studies (IVM), Vrije Universiteit Amsterdam, Amsterdam, The Netherlands; 6 Mediterranean Institute for Nature and Anthropos, MedINA, Athens, Greece; 7 NIBIO: Norwegian Institute of Bioeconomy Research, Ås, Norway; 8 Department of Land Management and Landscape Architecture, University of Agriculture in Krakow, Krakow, Poland; 9 Department of Geography, University of the Aegean, Mytilene, Greece; 10 Department Computational Landscape Ecology, Helmholtz Centre for Environmental Research-UFZ, Leipzig, Germany; 11 Geography Department, Humboldt University Berlin, Berlin, Germany; 12 Thünen Institute of Biodiversity, Johann Heinrich von Thünen Institute – Federal Research Institute for Rural Areas, Forestry, and Fisheries, Brunswick, Germany; 13 Institute of Geography, Slovak Academy of Sciences, Bratislava, Slovakia; 14 León, Spain; 15 Biodiversity, Agroecology and Landscape Management Lab (UMR BAGAP), National Research Institute for Agriculture (INRAe), Rennes, 35042 France; 16 Department of Geography, University of Latvia, Riga, Latvia

**Keywords:** Agricultural change, Driving forces, Green revolution, Land management history, Oral history interview

## Abstract

Farming in Europe has been the scene of several important socio-economic and environmental developments and crises throughout the last century. Therefore, an understanding of the historical driving forces of farm change helps identifying potentials for navigating future pathways of agricultural development. However, long-term driving forces have so far been studied, e.g. in anecdotal local case studies or in systematic literature reviews, which often lack context dependency. In this study, we bridged local and continental scales by conducting 123 oral history interviews (OHIs) with elderly farmers across 13 study sites in 10 European countries. We applied a driving forces framework to systematically analyse the OHIs. We find that the most prevalent driving forces were the introduction of new technologies, developments in agricultural markets that pushed farmers for farm size enlargement and technological optimisation, agricultural policies, but also cultural aspects such as cooperation and intergenerational arrangements. However, we find considerable heterogeneity in the specific influence of individual driving forces across the study sites, implying that generic assumptions about the dynamics and impacts of European agricultural change drivers hold limited explanatory power on the local scale. Our results suggest that site-specific factors and their historical development will need to be considered when addressing the future of agriculture in Europe in a scientific or policy context.

**Supplementary Information:**

The online version contains supplementary material available at 10.1007/s10113-023-02150-y.

## Introduction

Over the last century, most regions of Europe have experienced an unprecedented increase in agricultural productivity, through developments in plant and animal breeding, mechanisation, feeding, fertilisation and crop protection (Pellegrini & Fernández 2018; Jepsen et al. 2015; Gingrich et al. 2015). These changes have come at high environmental costs and raised serious concerns regarding the sustainability of agriculture (Tilman 1999; Kleijn et al. 2009; Campbell et al. 2017). In terms of ecological sustainability, research has often addressed the negative impacts on biodiversity, water quality and soil systems of changing land cover/landscape structure (van der Zanden et al. 2016; Tscharntke et al. 2021) and increased land use intensity, such as the use of more fertilisers, pesticides and heavier machinery (Prashar and Shah 2016; Keller et al. 2019). Developments in agriculture had also implications for economic and social sustainability (Diogo et al. 2022). These included, for example increasing income and gender inequality, eroding social cohesion and decreasing quality of life in rural communities (Kovačićek and Franić 2019; Maucorps et al. 2019; Augère-Granier 2017; Davidova and Thomson 2014). Yet, these aspects have been studied less (Janker et al. 2019).

Today, agriculture continues to be a central driver or subject of broad socio-economic and environmental megatrends and challenges. In the face of climate change, environmental degradation and population ageing, among other challenges, most European regions face increasing pressure to make transformational changes in agricultural practices (Debonne et al. 2022). The implementation of, and opportunities for, more sustainable practices are highly dependent on the regional and historical context, as well as the current agricultural land use system (Weltin et al. 2018). Studying patterns, processes and pathways of past changes is thus essential to gain insights into the factors that have shaped our current land use systems (Tappeiner et al. 2021). By understanding historical drivers, such as technological advancements, policy decisions and societal shifts, we can learn about the present, local option space for effective interventions to address ongoing sustainability challenges (Bürgi 2008) and to reduce potential conflicts of interests of local actors (Hernik et al. 2013).

Past development trajectories of today’s agricultural systems have been described using different approaches, such as studying long-term changes in land cover and land use (Fuchs et al. 2015; Kuemmerle et al. 2016) or analysing changes in intensity metrics (Erb et al. 2013; Plutzar et al. 2016). To study land use (intensity) change, top-down approaches are often applied, using, e.g. land use data from governmental or private statistical agencies (Levers et al. 2018; Schulp et al. 2019), geodata including information from remote sensing data and (historical) maps (Matasov et al. 2019; Pazur et al. 2021), or expert-informed narratives (Jepsen et al. 2015; van der Sluis et al. 2019). While such studies provide generalised insights, the classes of underlying drivers might not adequately reflect the actors’ perspectives (Plieninger et al. 2016). Conversely, actor perspectives on long-term land use change are typically assessed on a case study level. To obtain insight into farmers’ behaviour or to create farmer typologies on a larger scale, comparative case studies (e.g. Kristensen et al. 2016) or systematic literature reviews/meta-analyses (Malek et al. 2019; Bartkowski et al. 2022) have been conducted. However, such approaches tend to focus on the present situation, i.e. they hardly consider or analyse the historical trajectories of agricultural development.

To analyse the trajectory of past changes, some authors focus on points in time, such as chains of events (Walters 2017) or leverage points leading to regime shifts (Müller et al. 2014; Fischer and Riechers 2019), while others focus on longer periods, for example by studying legacy effects (Munteanu et al. 2015; Tappeiner et al. 2021) or stagnation and inertia in reinforcing systems (Zariņa 2013). Further approaches seek to better understand causality in land use and landscape change (Meyfroidt 2016). However, land use systems are often very complex, involving multiple actors and various spatial, temporal and institutional scales (Schneeberger et al. 2007). The concept of driving forces (DFs) is often used to obtain a comprehensive overview of the factors contributing to landscape change (Plieninger et al. 2016; Bürgi et al. 2017) or land system change (van Vliet et al. 2015). Bürgi et al. (2004) distinguished between natural, technological, cultural, economic and institutional DFs. A similar set of classes is used in most DF studies, with slight differences in taxonomy or number (van Vliet et al. 2015; Plieninger et al. 2016; Jiménez-Olivencia et al. 2021).

In this paper, we examine the main DFs of farm change in European farming systems since the 1960s from the farmer perspective. In contrast to other studies that have analysed temporal dynamics (Bürgi et al. 2017; Jepsen et al. 2015), we do not attempt to distinguish different periods, but search for pattern in DFs as perceived by farmers overall. By shifting the focus to the farmers, i.e. one of the central actors in most agricultural systems, we aim to (a) gain insights into historical DFs from an underrepresented perspective and (b) compare perceived driving forces between spatially and thematically diverse study sites. To achieve a long-term actor-oriented understanding of DFs of farm change, we conducted oral history interviews (OHIs) with experienced farmers who were either retired or nearing retirement in a comparative study with 13 study sites spread across 10 European countries. Guided by a DF framework (Bürgi et al. 2004), we analyse the interviews for the DFs mentioned for overall farm change.

Specifically, we ask the following research questions:

1. What are the DFs in the rationale farmers give when explaining changes or intentional persistence on the farm?
2. How (dis-)similar are the DFs between the study sites and what causes these patterns?

Changing perspective also means looking at farm change through the eyes of practitioners and interpreting farm change in terms of all aspects of a farm, recognising that a farm is an economic enterprise that exists through the engagement of a single person/family/company.

## Material and methods

OHIs are a type of source in historical studies used to capture history not recorded in standard sources or to supplement them (Schaffner 2013; Mohr et al. 2023). The focus is often on everyday history, labour history or generally on the experiences of population groups that have historically not been perceived as worthy of documentation (Wierling 2003). Recently, OHIs have also been used more frequently in land use and landscape science to study changing land use practices or other reasons for landscape change (Wunderli 2016; Li et al. 2017; Bürgi et al. 2017). To conduct and transcribe the OHIs in different languages, we established a network of academic partners (= study site partners), who organised and/or conducted the interviews. They also provided in-depth background information needed to locate the study site or prepare for the interviews and reflected the results of the driving forces analysis for each study site.

### Study sites

**Fig. 1:**
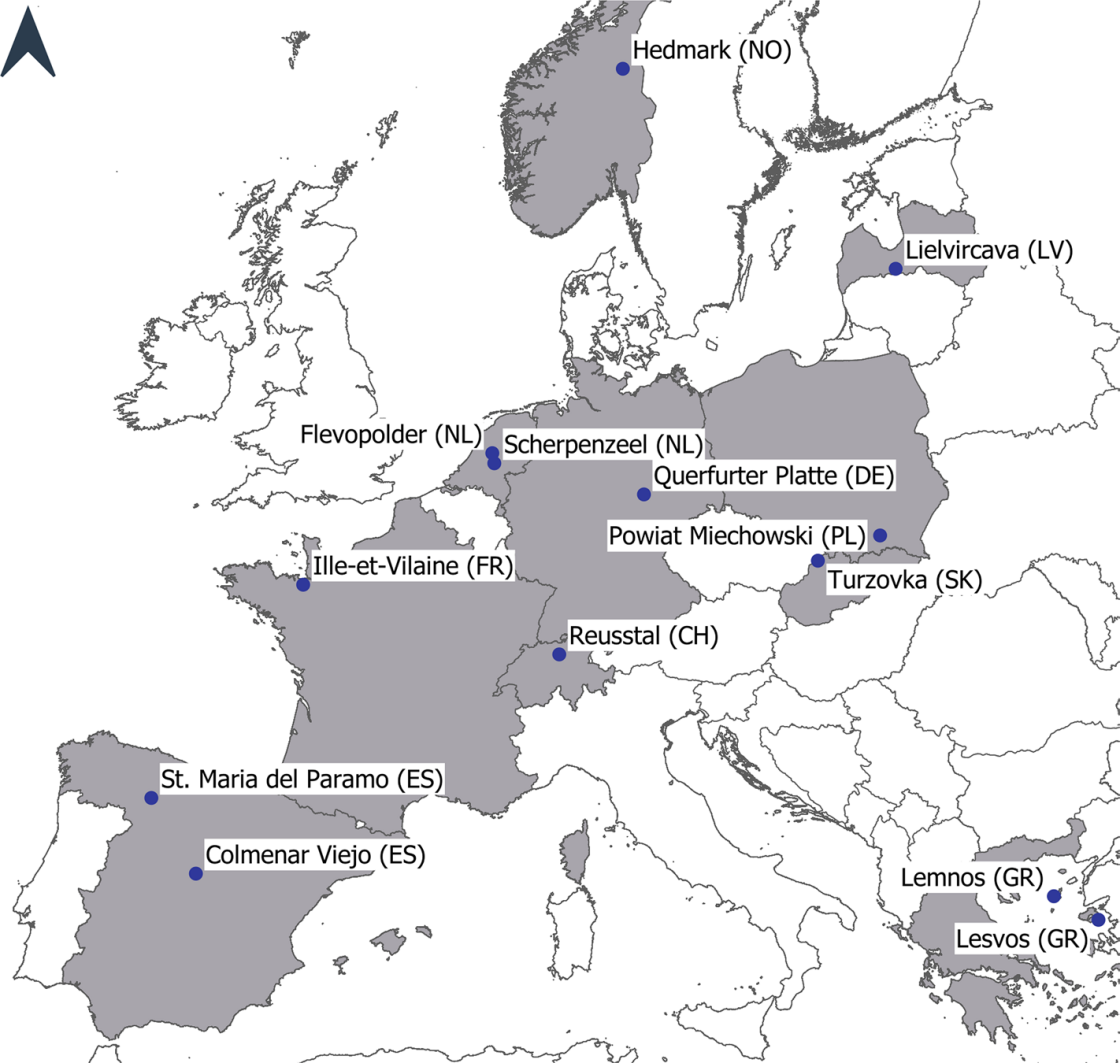
The 13 study sites are spread over 10 European countries and cover a wide range of environmental, socio-cultural and agricultural contexts

We selected the 13 study sites to span a wide array of land system characteristics, climatic conditions and institutional/political systems so that the case study set as a whole is representative of the broader European context (Fig. 1, Table 1). We started with a list of set of study sites where local contacts had been established from previous studies (e.g. Herzog et al. 2006; Bürgi et al. 2017). We then assessed the geographic representativeness of each site in relation to the European context. This was done by computing spatial similarity index maps indicating the degree of statistical similarity, evaluating the overall representativeness of the study sites and identifying still underrepresented regions (see Diogo et al. 2023 for detailed procedure). In each study site, we delineated an area of 5 × 5 km^2^ that encompassed similar farming systems within the same landscape.

**Table 1:** Overview of the study sites

Site name	Country	Environmental zone^a^	Farm type(s)	Number of interviews
St. Maria del Paramo	Spain (ES)	Mediterranean	Arable crops; one singular sheep/dairy farm	10
Colmenar Viejo	Spain (ES)	Mediterranean	Dairy farming and meat production; little fodder production	10
Lemnos	Greece (GR)	Mediterranean	Sheep farming (for milk and meat); arable crops and grasslands	11
Lesvos	Greece (GR)	Mediterranean	Olive trees; some animals for own use	10
Querfurter Platte ^b^	Germany (DE)	Continental	Large-scale arable crops; a few mega stables	11
Turzovka^b^	Slovakia (SK)	Continental	Mix of large-scale livestock farms and small ‘hobby’ farms	7
Powiat Miechowski^b^	Poland (PL)	Continental	Mixed farm systems	10
Lielvircava^b^	Latvia (LV)	Boreal	Arable crops; a few livestock farms and orchards	8
Ille-et-Vilaine	France (FR)	Atlantic	Dairy farming and meat production; arable crops and grasslands	9
Scherpenzeel	Netherlands (NL)	Atlantic	Livestock farms; little arable farming for fodder production	7
Flevopolder	Netherlands (NL)	Atlantic	Arable crops; some organic farms	10
Reusstal	Switzerland (CH)	Continental	Livestock farms, with dairy farms dominant; arable crops and grasslands	10
Hedmark	Norway (NO)	Boreal/alpine	Dairy farming and meat production; predominantly grasslands, some arable crops	10

^a^According to LANMAP (Mücher et al. 2010)

^b^These sites experienced a socialist system, which strongly influenced the development of agriculture through planned economy, socialist ideologies and the collapse of socialism in the late 1980s/early 1990s

The study sites selected include regions dominated by arable farming as well as livestock-oriented agriculture. In some of the livestock-oriented regions, agricultural activities also include arable crops and grassland, whereas others depended foremost on imported fodder (see Table 1 for a detailed description of the farm types). A special case is Lesvos (GR), where the main agricultural activity is the production of olives. While most of the study sites are intensively farmed, some are currently affected by abandonment—especially in hillier, less accessible areas (Turzovka (SK), Lemnos (GR), Lesvos (GR)). From the end of the Second World War until around 1990, the study areas of Querfurter Platte (DE), Lielvircava (LV), Powiat Miechowski (PL) and Turzovka (SK) were governed by a socialist regime. This led to the collectivisation of farms and the introduction of a planned economy, except in Powiat Miechowski (PL), where family farms remained but became part of the planned economy. Flevopolder (NL) is located in a part of The Netherlands that was only reclaimed from the sea in the 1960s. Except for Reusstal (CH) and Hedmark (NO), all study sites are in the European Union (EU) and subject to the Common Agricultural Policy (CAP). However, some of the study sites have been part of the EU (or its predecessor) for longer (e.g. Flevopolder (NL), Ille-et-Vilaine (FR)), while others joined only relatively recently (e.g. Lielvircava (LV), Powiat Miechowski (PL)). The diversity of the study sites is also reflected in the farm characteristics, such as the farm size, which ranges from a few hectares for hobby farmers in Turzovka (SK) or olive farmers in Lesbos (GR) to 6500 ha for a cooperative farm in Querfurter Platte (DE) (for further information on the individual study sites, refer to the study region portraits in Appendix I). Younger/still-active farmers in the same study sites were interviewed in a complementary process to determine current practices and agricultural development pathways (Helfenstein et al. in review).

### Oral history interviews

#### Target group and interview characteristics

**Fig. 2:**
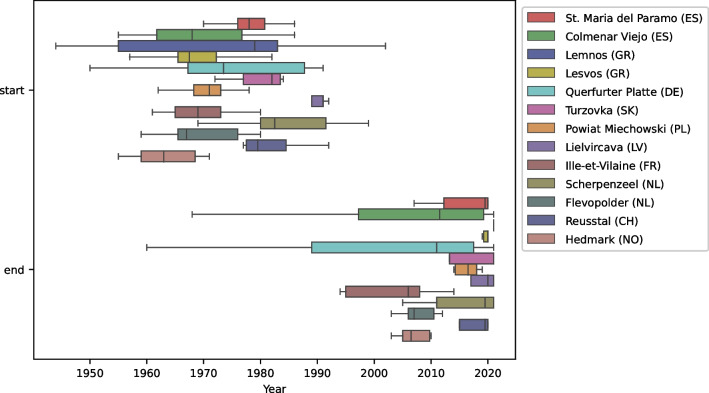
The time periods covered by the oral history interviews (OHIs), visualised with a box plot for both the start point (top) and the end point (bottom) for each study site. Outliers are excluded. The order of the box plots is as indicated in the legend

The target group was experienced farmers who were either already retired or close to retirement, in order to gain insights into the long-term development of farms. This allowed us to collect information from the 1960s onwards (Fig. 2). The interviewees were selected using the snowball method (Leavy 2017). In certain study sites, interviewers could build upon a pre-existing network, while in others we made first contacts by going door to door, finding farms on Google Maps or reaching out to farming or historical associations. Between May 2020 and August 2021, we conducted between 7 and 11 interviews in each study area, adding up to 123 OHIs in total. In many of the study sites, traditional gender roles were still evident for the generation interviewed, with the man in charge of the farm and the woman either looking after the household and providing selective assistance on the farm or supporting the family through off-farm work. Consequently, we carried out only 20 interviews with women and 27 interviews with more than one person (husband and wife; two generations), most of them in Ille-et-Vilaine (FR), Flevopolder (NL) and Hedmark (NO). Due to the COVID-19 pandemic, the interviews in Scherpenzeel (NL) had to be conducted by telephone. We conducted all other interviews face-to-face. On average, interviews lasted about 70 min (SD = 38 min); however, this varied considerably due to different conversation cultures, availability and interview settings.

#### Questionnaire

We designed a semi-structured questionnaire to address the target group appropriately (with open-ended questions, not survey scales) and to overcome the double bind of allowing freedom for site-specific responses, thus understanding the background of agricultural change, while still making the interviews comparable between the study sites. The questions focused on the period when the interviewee was active on the farm and involved in the main farm decisions. For interviews with active farmers, we used the year of the interview as the end point (2020/2021). As a result, the number of years discussed in the interviews varied (Fig. 2). For collectivised farms/cooperatives, we combined the information from interviewees working on the same farm (Lielvircava (LV) and Querfurter Platte (DE)). Kolkhozes, which became, e.g. cooperatives after socialism ended, were considered as one farm for the whole duration.

The questionnaire (Appendix II) was divided into two parts. The first part was about the interviewee’s personal experience working in agriculture, including topics such as life stages and social and economic aspects. The second part was about changes on the farm (e.g. farm size, fertiliser use) and the corresponding reasons. The questionnaires were translated by local experts into the language of the study site. The interviews were recorded and afterwards transcribed and translated into English. There were two exceptions to this procedure: in Powiat Miechowski (PL) notes were taken directly during the interview, and in Hedmark (NO), a detailed summary was written for each question based on the recordings. Both the procedure and the questionnaire were approved by ethical clearance from the Ethics Commission of the Swiss Federal Institute of Technology (ETH-EK 2020-N-146) and a prior informed consent was obtained from all respondents.

### Analysis of oral history interviews

#### Conceptual background: driving forces framework

To structure the variety of aspects that were mentioned for the transformation of the farms, we followed the DF framework proposed by Bürgi et al. (2004), in which DFs are divided into five categories (Table 2) that are seen as external forces that induce change. DFs can originate from different scales and can co-occur. During the process of coding and creating subcodes, we adjusted some of the original categories to adapt them better for the OHI data (see below).

**Table 2:** Description of the main driving forces (DFs) categories considered in this study. Bold font indicates terminology as used by Bürgi et al. (2004); normal font indicates adjustments made for this study to better reflect the oral history interview (OHI) material

Main DFs categories	Exemplary topics
**Cultural** & personal	Changes in demography on the municipal scale or intergenerational arrangements on the farm level; also includes socio-cultural aspects like cooperation
**Economic**	Change in price; access to markets; strategic decisions to increase the farm size (e.g. land, animals) to react to structural change
**Institutional**	Political changes such as socialism; foreign relations; agricultural policy including the introduction of subsidies; land consolidation
**Natural **& spatial	Location factors such as water availability or soil quality; temporal influences like pests and pathogens or weather events
**Technological**	Introduction or development of new technologies, such as field machinery, irrigation system, fertiliser

#### Developing the coding book

**Fig. 3:**
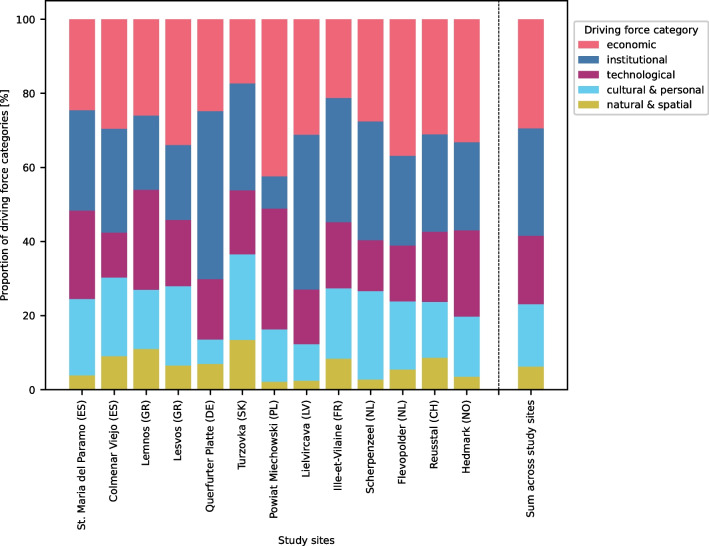
Prevalence of driving force (DF) categories in the different study sites. Economic and institutional DFs were generally the most frequently mentioned, followed by technological, cultural & personal and natural DFs. The strength of the DF categories is determined by the number of detailed DFs specified per category and how frequently it was mentioned in the oral history interviews (see Appendix IV)

Based on the conceptual framework, we coded the transcripts of all 123 interviews into the main DF categories (Table 2, Fig. 3). We defined statements as DFs when they indicated (a) a specific change at the farm level or a consciously chosen persistence and (b) an attributed cause or motivation for the respective change, e.g. ‘So in the beginning we had different types of grain: wheat, oats, barley. And later that was limited to wheat because the oats and barley didn’t yield anything’ (Flevopolder (NL), f8). In a few cases, we extended this rule to statements that did not contain both elements, but where evidence could be clearly inferred from the previous and following statements.

In a second step, we inductively formed thematic subcodes, i.e. more specific DFs, in iterative steps based on the coded segments following the concepts of qualitative content/thematic analysis (Braun and Clarke 2006; Erlingsson and Brysiewicz 2017). We chose subcodes that were as general as possible and as specific as necessary, so that they could be applied to more than one study site but still capture the characteristics of each study site. Through an iterative process, we defined a total of 68 subcodes and used them to code all interviews a second time (see Appendix III for an overview and short description of all subcodes). In contrast to other studies (Bürgi et al. 2017; Helfenstein et al. 2020), we included not only external demographic and societal factors as subcodes for the category of cultural DFs, but also more personal reasons for changes on the farm, such as perceived good practices and personal preferences/attitudes. Consequently, we renamed the category as ‘cultural and personal DFs’.

In some cases, subcodes from different categories of DFs were highly interrelated, such as the need to optimise labour and the introduction of labour-saving technology, for which subcodes from both economic and technological DFs were plausible. In such situations, we chose the DF that the farmers themselves mentioned, to reflect their specific perception. This procedure resulted in different numbers of subcodes per DF category, as well as different frequencies of the subcodes across the different study sites. We discussed the resulting selection of subcodes for each study site with the corresponding study partners before developing the overall analysis.

#### Further processing of subcodes

To allow for a comparative perspective across all study sites, we structured the 4418 subcode quotes from the 123 interviews across the 13 study sites based on vote counts. While we are aware that the use of counts is controversial among scientists working with qualitative interviews (Sandelowski 2001; Hannah & Lautsch 2011), we considered this a necessary step to uncover broader patterns. Nevertheless, we do not consider the number of counts to necessarily reflect the absolute significance of a subcode. When an interviewee mentioned a subcode several times, we counted it only once. We only included subcodes in our analysis that were mentioned more than once per study site and at least six times across all study sites. As the number of interviews varied between study sites (Table 1), we normalised the absolute counts to percentages to allow comparisons between study sites. To check for correlations between the study sites, we calculated Spearman’s rank correlation coefficient.

To identify subcodes with high relative importance across all study sites, we calculated the sum of mentions across all study sites for each subcode and extracted the ten subcodes with the largest sums. Next, we highlighted subcodes that had many mentions in individual study sites but ranked low in the relative importance across all study sites (see Appendix IV for details). We expected that these subcodes would be more contextual and thus allow the derivation of dominant site-specific DFs. We visualised the relative importance of the subcodes with heat maps for both subcode sets, overall and site-specific importance. While the term ‘subcode’ is useful for describing the methodology, we rather use the term (more detailed/specific) DFs in the following sections.

## Results

### Perceived driving forces of change in agriculture

When expressed in relative terms, economic and institutional DFs were the most frequently mentioned across all sites, followed by technological, cultural and natural DFs (Fig. 3). Even though there are fewer detailed economic DFs specified than institutional DFs, these two categories received similar numbers of overall mentions (see Appendix IV). Technological and cultural DFs received similar numbers of mentions with an equal number of more detailed DFs specified, while natural DFs score the lowest in both analyses. While most study sites follow this general trend, there are some study sites that deviate from it. For example, in Powiat Miechowski (PL), economic and technological DFs were mentioned more frequently than the average, while institutional DFs were rarely mentioned.

**Fig. 4:**
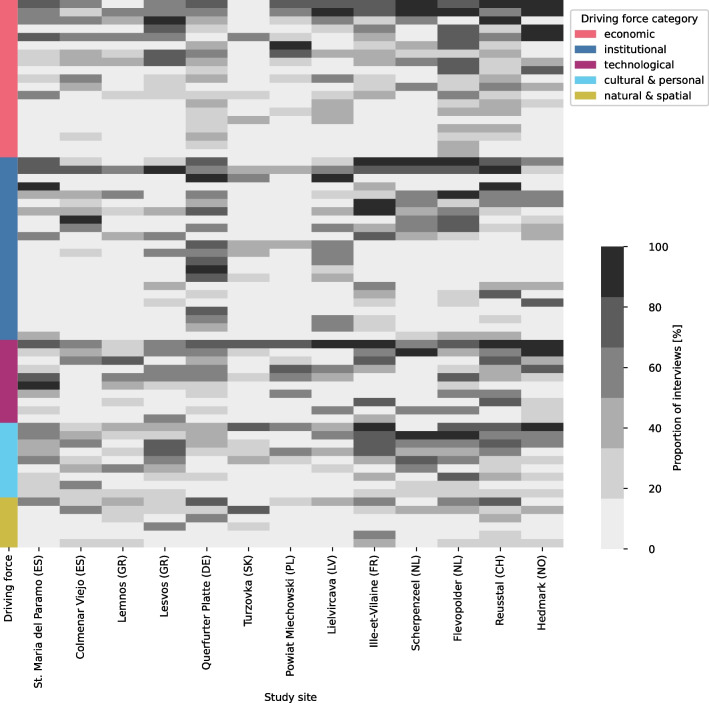
Diversity of the study sites, expressed in terms of the number and frequency of the more detailed driving forces (DFs = rows) identified per DF category. Dark grey means that nearly all interviewees in the study sites mentioned the DF, while light grey indicates fewer mentions. The abundance of all DFs (rows), the associated DF categories (leftmost column) and the prevalence of the individual DFs (shades of grey) across the different study sites (remaining columns) are depicted. A list of all identified DFs with a general explanation can be found in Appendix III

Regarding the abundance of more specific DFs and their varying mentions across the study sites (Fig. 4), each study site shows a distinct ‘barcode’ (Fig. 2 of Appendix IV)). The highest correlations in these profiles appear between Scherpenzeel (NL) and Colmenar Viejo (ES), both being intensive livestock systems with farmers largely depending on feed imports (Spearman rho: 0.63), Querfurter Platte (DE) and Lielicarva (LV) that share a (post-)socialist history (Spearman rho: 0.62), and Reusstal (CH) and Ille-Vilaine (FR) that mostly specialised on dairy cows or/and beef cattle, while producing much of the feed themselves (Spearman rho: 0.62). Within each main category, some DFs were mentioned very frequently in most study sites, while others were mentioned frequently in only a few study sites, indicating specific regional configurations. In the following sections, we present these two groups of DFs separately.

### Driving forces with a high prevalence across all study sites

**Fig. 5:**
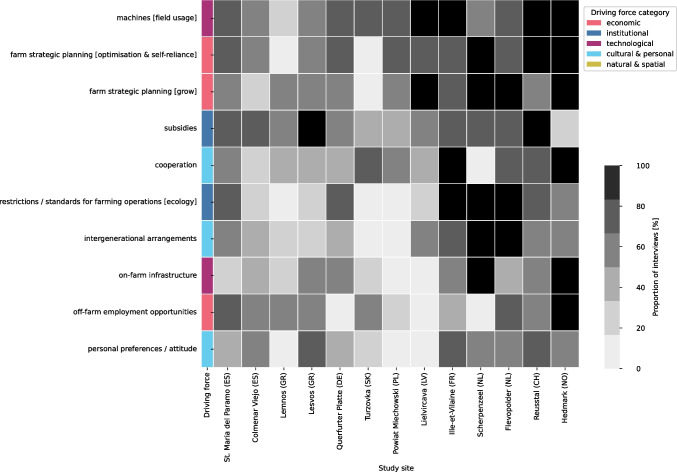
The ten driving forces (DFs = rows) that ranked highest across the study sites (columns). The DFs shown are categorised into technological, economic, institutional and cultural & personal DFs. Dark grey means that nearly all interviewees in the study sites mentioned the DF, while light grey indicates fewer mentions

The ten DFs most frequently mentioned across all study sites (Fig. 5) include DFs from all categories except natural DFs. Apart from the cultural & personal DFs, all DFs are either related to the modernisation of the agricultural system (technological DFs) and the associated increase in scale while reducing labour (economic DFs) or to agricultural policy instruments (institutional DFs).

The most frequently mentioned technological DF was ‘machines [field usage]’, which allowed farmers to reduce labour and/or cultivate more land: ‘They mow at the moment with a grass machine that is 9 m wide. […] I was mowing the lawn with the horse, now look at that. It wasn’t 9 m wide, it was just 1 m wide. Just look how long it took to mow one hectare. Now they do it in 10 min’ (Scherpenzeel (NL), f6). The most commonly mentioned field machines across study sites were tractors, while combine harvesters, seed drills and chainsaws held specific importance in some study areas. ‘On-farm infrastructure’, such as silos, loose housing or fencing, also played an important role in regions dominated by livestock farming, while in Lesvos (GR), olive collection nets were an important additional infrastructure introduced to speed up olive harvesting.

The two most frequently mentioned economic DFs are not related to individual factors, but to a combination of external economic developments and farmer’s intrinsic motivations, often leading to changes in farm orientation and organisation (= ‘farm strategic planning’). External economic developments include, for example, changes in commodity market prices, average per-unit production costs and average productivity. Intrinsic motivations are related to the willingness of the farmer to, for example be self-sufficient, keep the farm economically viable or expand operations. External economic developments and farmer’s intrinsic motivations can interact in self-reinforcing ways, for example leading to broad structural changes in the agricultural sector over time, such as increasing average farm size (e.g. overall increase of the area under cultivation and/or the number of livestock/trees) and decreasing number of farms. Another response is to optimise work processes and resources. This DF is closely linked to technological aspects, but the emphasis is on the need/opportunity for better machinery/work organisation to be competitive: ‘[We bought the machines] to increase efficiency. Just work. […] And the problem is that everything has to be done in a short time. It has to be done quickly’ (Reusstal (CH), f6). Off-farm employment opportunities were also of importance, if adapting the farm strategy to structural change and market demands was not possible: ‘You have to choose either to grow bigger or to do something else. If you line up 10 farms, there are eight that do something else on top. Or the farmer’s wife has a good job, and things like that. Care farms are being set up. Things are done on the side’ (Flevopolder (NL), f9).

On the institutional side, the introduction/adaption of subsidies and direct payments for farming operations, as well as standards and restrictions, were mentioned. These DFs sometimes occurred in combination, for example when the eligibility for direct payments was linked to the implementation of restrictions and standards. Both the specific subsidies for a certain product and the change from product support to direct payment were widely mentioned, with farmers often expressing their economic importance: ‘That is not negligible! Without these sums [direct payment], no farm in Europe could exist. […] If the farms, whether they are larger or smaller farms, had the possibility to produce at corresponding market prices, we wouldn’t want these EU subsidies. Because there you always have us on the leash’ (Querfurter Platte (DE), f5).

Three cultural DFs were mentioned frequently. First, cooperation was identified by many farmers as a necessary base condition to run a successful farm, especially in the past until full mechanisation replaced it, which was often noted with regret: ‘I think that before there was much more union than nowadays. […] If you had to lend a hand to others or if you needed to be helped, there was solidarity in that sense. Also, there were groups of three or four people who joined and worked together’ (Santa Maria del Paramo (ES), f3). In Hedmark (NO), a very remote and alpine site, cooperation is still essential for the farming community today. Second, the issue of intergenerational arrangements for farm transfer was an important factor, especially for family farms, but it was also mentioned with regard to cooperative farm/agribusiness (Querfurter Platte (DE), Lielvircava (LV)). While the existence of a successor was mostly perceived as positive, in some study sites (Ille-et-Vilaine (FR), Scherpenzeel (NL), Flevopolder (NL), Colmenar Viejo (ES)) some parents actively discouraged their children from taking over the farm because they did not want them to go through the same hardships they had experienced or because they considered the farm too small to remain viable. Finally, personal preferences and attitudes also played a role in farm decisions, such as the choice of cow breed based on personal taste or the voluntary decision not to carry out certain activities to ensure the farm remained environmentally friendly.

### Site-specific driving forces

**Fig. 6:**
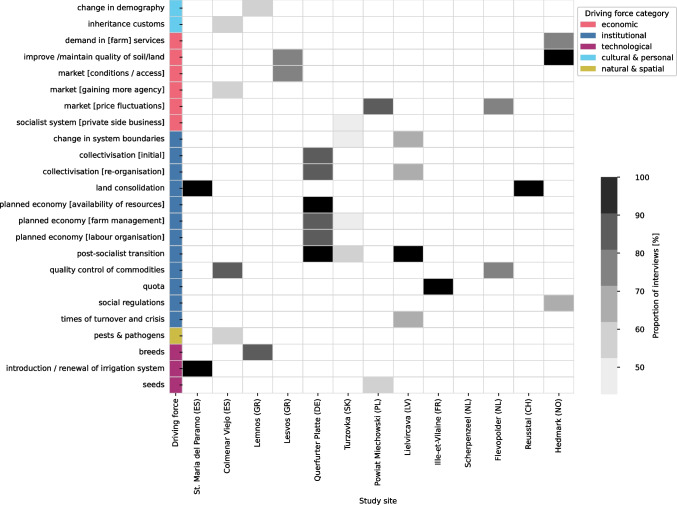
For each study site, driving forces (DFs) were selected that rank high in the corresponding study sites, but not overall. They reflect site-specific DFs. Dark grey means that almost all interviewees in the study sites mentioned the DF, while light grey indicates fewer mentions. Values that did not meet the filtering criteria (see Appendix IV) are shown in white

In addition to the commonalities between the study sites, DFs with a more singular significance were also identified for all study sites except for Scherpenzeel (NL). These fit well into the broader, site-specific narrative (Fig. 6).

For both Reusstal (CH) and St. Maria del Paramo (ES), the DF referring to a large-scale land consolidation project at the communal level ranked very high. Through land consolidation, farmers received fewer but larger plots, enabling more efficient farm management that increased the competitiveness of the farms: ‘It was good that we then had the land closer together and larger areas. One could then work more efficiently’ (Reusstal (CH), f4). This reorganisation of the land came along with the removal of trees and hedges and in both study sites it also included measures for improving the land. For Reusstal (CH), this meant more efficient drainage of the former wetlands, fewer field trees and additional layers of humus, while for St. Maria del Paramo (ES) it meant optimising the land for a large-scale, community-spanning irrigation system that made intensive large-scale farming possible.

In Querfurter Platte (DE), DFs relating to the socialist regime, as well as measures after the reunification of Germany, were mentioned by many respondents. Between 1946 and the 1970s, agriculture underwent a radical transition as family farms were first collectivised and then industrialised (advances in machinery, separation of animal and plant production, fields up to 150 ha), resulting in so-called Agricultural Production Cooperatives (i.e. Kolkhozes) of up to 7000 ha. With the collapse of socialism, the region underwent another major change, with Agricultural Production Cooperatives either being broken up and taken over by resettled farmers and newcomers or having to adapt to a capitalist economic system. Many of the large-scale structures (e.g. farm size, field size, mega stables) that were started during the socialist period have remained until today. The site-specific, dominant DFs in Lielvircava (LV) were also shaped by socialist management, as well as by the policies of the transition to post-socialism. The transition was followed by a turbulent period in the early 1990s, during which farmers experienced a regulatory gap that led, for example to market fraud. Formal agricultural education, or the lack of it, was also a central topic mentioned in Lielvircava (LV), due to newcomers after the land reform around the breakdown of the socialist system. Market fraud and (lack of) formal education both had a direct impact on farm viability/success, especially for smaller farms. Turzovka (SK) was influenced by collectivisation that took place in the main valley, while small, marginal farms on the valley slope continued to operate. However, the owners of such private farms were working fulltime in nearby factories and used the land to grow food for themselves and to earn additional money by breeding bulls for the Kolkhoz. The small, marginal farms often remained ‘hobby-farms’ after the collapse of the socialist system and were gradually abandoned, while the valley floor is still dominated by large farms today. Powiat Miechowski (PL) is located in a region of Poland where collectivisation was not enforced, and family farms persisted. In this study site, the uncertainty and fluctuation of prices caused a lot of insecurity, which affected the economic viability of the farm; on the other hand, new crop varieties helped to increase production and thus economic viability.

Although a milk quota was introduced in most of the study sites, its impact was most strongly mentioned in Ille-Vilaine (FR), where it coincided with the start of specialisation and modernisation on many farms. The quota had an impact on farm strategy possibilities of not yet fully developed dairy farms with many farms diversifying their strategies and some stopping in the next generation. In Colmenar Viejo (ES), site-specific DFs were mostly related to the general situation in the 1980s—around the time of Spain’s entry into the European Economic Community (EU predecessor). This period saw the introduction of new hygiene standards, in particular hygiene measures for milk production, stricter rules for direct marketing and a reduction in the price of milk. The introduction of milk testing led to the discovery of tuberculosis and brucellosis in many cow herds, resulting in (sometimes repeated) mass culls. As this was combined with a low milk price and new subsidies for beef cows, some farmers stopped having dairy cows and switched to meat production. The remaining dairy farmers chose to strengthen their position by setting up a dairy cooperative in 1985 to gain more agency in the market and thus better conditions.

The two Greek study sites are located on islands with similar climatic and biophysical conditions, but the land use system and the site-specific DFs are different with olive trees on Lesvos and livestock and crop farming on Lemnos (see Dimopoulos et al. 2023 for details). Until the 1950s, there were many small farmers in Lemnos (GR), which meant that land was scarce. Many islanders emigrated in the following decades, mainly because there were more economic opportunities elsewhere, leaving more land for those who stayed. The influence of breeds was another key issue, because after a long period of using mainly Lemnian sheep breeds, farmers began to import breeds, first from Lesvos, then from Germany and France. This led to an increase in production, but also to more vulnerable flocks and economic sorrows in case of disease. In Lesvos (GR), a fall in the price of olive oil relative to the cost of living was one of the most pronounced DFs and was generally seen as the cause of de-intensification, problems finding successors and the abandonment of less profitable olive groves. When it was still profitable to grow olives, it was important to build and maintain terraces to increase the area that could be grown, while limiting soil erosion and retaining water.

Site-specific dominant DFs for Hedmark (NO) were related to increasing the income of the current farm. On the one hand, to increase grass production, additional, mostly wooded land was cultivated, drained and sometimes levelled to make it usable for machinery. On the other hand, farm services, such as farm stays in the 1980s and 1990s, or forestry more recently, were opportunities to earn some money in addition to the income from what the farm could produce.

In Flevopolder (NL), many farmers talked about private quality control companies that they de facto had to use to have certified commodities, which are especially demanded by international companies. This meant additional costs for the farms, as well as a lot of time spent filling out forms and dealing with inspectors. Fluctuations in crop prices also led many farmers to diversify their production, combining some stable with some riskier products.

## Discussion

### Added value of studying farmer perceptions and insights gained

Our analysis reveals that several DFs were considered important in shaping agricultural development across very different farming contexts within Europe, but that at the same time the relative importance of DFs differed largely between study sites. These results contrast to previous studies, which either identified specific DFs for a small geographical area based on case-study evidence (e.g. Hersperger and Bürgi 2009; Eiter and Potthoff 2016; Vinogradovs et al. 2018), or provided a summary of the general impacts of DFs using large-scale approaches or systematic literature reviews (e.g. van Vliet et al. 2015). Our systematic, comparative case-study allowed investigating long-term, site-specific farming realities without being absorbed by the singularities occurring in the specific study region selected. For studies mapping DFs across larger regions up to the European scale, the set of DFs considered has to be selected a priori based on conceptual considerations and data availability (e.g. Plieninger et al. 2016; Levers et al. 2016; Matasov et al. 2020)—which may not correspond to the perspective of local actors. The use of a comparative study design combined with OHIs allowed us to start with local narratives and subsequently project them into a system of more generally valid DFs.

In many systematic literature reviews (e.g. van Vliet et al. 2015; Plieninger et al. 2016), natural DFs, sometimes also referred to as location factors, are given a prominent place. While natural DFs/location factors play a central role in the possibilities for different farming styles, our results suggest that they are also a given reality for local actors that is not subject to substantial change and therefore are not central to the interviewees’ rationale for farm development. It appears that the study design largely determines how (natural) DFs are weighted. The OHIs from the Querfurter Platte (DE) gave an interesting indication when farmers mentioned the increasing frequency of droughts in summer and the resulting crop losses: ‘And the farms, both private and cooperative, now live off these reserves. Because with these harvests, with this drought, nothing is coming out’ (Querfurter Platte (DE), f1). Irrigation is not viable, due to the hydro-geological characteristics, so it is plausible that for this site—as for others (e.g. Debonne et al. 2022) —climate change is likely to increase the importance of natural DFs in the rationales as farmers adapt to the new conditions.

Whereas some of the cultural and personal DFs have also been highlighted in other studies (e.g. intergenerational arrangements with focus on succession for farm characteristics in van Vliet et al.), cooperation is less frequently mentioned in the conventional analysis of DFs. While there is case-study literature recognising the potential importance of cooperation (de Roest et al. 2018) or studying cooperation between farmers directly (Aurbacher et al. 2011; Emery 2015), there is little focus on it in more generalised analyses, probably because cooperation is not captured in agricultural statistics and is not easy to measure over larger areas. It may therefore be relevant to further explore how a resumption of cooperation between farmers could emerge in today’s world to improve the different dimensions of agricultural sustainability. As Leventon et al. (2017) found, collaboration, and in that sense also cooperation, can strengthen the effectiveness of CAP biodiversity measures, but the current subsidy system misses such opportunities through a focus on individual actions.

Comparing different studies with similar foci reveals the extent to which research design influences which DFs can be addressed and evaluated. In a long-term study (1800–2010) of European land management, Jepsen et al. (2015) collected and synthesised information on DFs based on national narratives. A comparison of the DFs identified in this study (Appendix III) with the concurrent periods/regimes of Jepsen et al. (2015) reveals two main differences. While the technological DFs determined by Jepsen et al. (2015) were largely also mentioned by farmers, our approach additionally brings up topics such as innovations in farm infrastructure (e.g. silos or new types of stables), additives (pesticides, antibiotics) and new/evolving breeds/seeds as technological DFs for some of the study sites. Both studies also show a high correspondence in economic and institutional DFs. However, the farm perspective additionally reveals more subtle aspects of these main DFs. For example, while Jepsen et al. (2015) identified world market integration as the main economic driver for a period overlapping with our study period, the farmers interviewed, elaborated on changes in market prices or having to reorganise their farm (i.e. farm strategic planning) due to the changing economic realities, which are arguably farm-level consequences of global integration.

### Do driving forces act in bundles?

Many DF studies focus on the identification of DFs and their respective and often combined importance for land use and landscape change processes (van Vliet et al. 2015; Plieninger et al. 2016; Daunt et al. 2021). The richness of the OHI data collected for this study provides more detailed insights into DFs that had particular large effects because they seemed to co-occur in bundles.

We observed that many agricultural policies, such as the introduction of dairy quotas, were theoretically applicable/valid across many study sites, but the specific relevance for farm development, depended on, e.g. farm structure and size. In Ille-et-Vilaine (FR), the introduction of milk quotas was perceived as stopping overall yield growth, with farms that were already highly productive being able to maintain a high level of production. At the same time, it became impossible for smaller farms to grow and specialise in milk production alone, leading to diversification (e.g. additional meat production, tobacco growing) or discontinuation of these farms: ‘But you see, when I set up, there were 153 farms in the municipality. When I stopped, there were 20. So, because of the milk quotas, in the area, a lot of people were growing potatoes and were not developed in milk, and then they stopped growing potatoes, but there was no more development in milk [possible]. So, they have felt the full impact of the quotas. Whereas the larger farms that were already oriented towards milk were more at ease’ (Ille-et-Vilaine (FR), f6).

An example of more general co-occurring DFs—also reflected in Fig. 5—is the uptake of new technological inventions (e.g. tractor, combine harvester or precision seed drill), the decrease of commodity prices and the economic need to optimise labour and resources and/or to increase the farm size. The circular re-enforcement of these driving forces is sometimes referred to as the technological treadmill (Ward 1993), which pushes farmers to increase production with ever newer and more efficient technologies, leading to higher production and thus lower commodity selling prices, which in turn pushes farmers to further optimise their production. This combination of technological and economic drivers is often mentioned in the context of a productivist mindset that aims to maximise production (Wilson 2001), increasingly promoted from the 1960s onward. While government programmes initially supported this attitude, since the 1990s, in the face of negative sustainability impacts and overproduction, agricultural policies have tried to limit it, for example by introducing direct payments per hectare (instead of market support) or by regulating the use of fertilisers (Ilbery and Bowler 1998). However, challenges remain in agriculture, as economic realities continue to force farmers to scale up and specialise (Abson 2019) and the CAP struggles to meet sustainable development goals (Pe'er et al. 2019).

These two examples show that while it is important to analyse the distribution and strength of the identified DFs, it is also critical to recognise that these DFs often only have a major impact on farm change when they appear in certain constellations, i.e. as bundles. It is important to consider such intertwined effects of policies for different regions and their effect on farm development in order to better align policies with intended outcomes. Future studies may benefit from examining whether the same bundle of driving forces is responsible for the similar change in different regions or whether the effects are related to land use systems.

### Methodological consideration and source critique

While OHIs are an important tool to give actors a voice alongside standard sources when analysing past changes (Schaffner 2013), it is also important to reflect on the associated limitations and strengths. Although the interviews were not completely open, but based on a semi-structured questionnaire, this format allowed for much more personal conversations with the interviewees compared to asking simple closed questions. However, this also meant that the character of the interviews was influenced by the interview culture and the intentions of both the interviewer and interviewee, as well as the selection of interviewees (e.g. position, gender). While a closed-ended questionnaire approach would have increased the comparability of results, the selection of possible answers to why farm changes have occurred would have been more strongly shaped by the a priori knowledge and biases of the researcher (see also Added value of studying farmer perceptions and insights gained). Coding the transcripts by classifying the text into the inductively generated DFs for the standard categories allowed us to gain a systematic understanding, but also involved deconstructing themes that were actually raised together. Instead of coding individual DFs, another approach could have been to use narratives (Bürgi et al. 2017; Rois-Díaz et al. 2018) or storylines (Frei et al. 2022) to illustrate the DFs for the individual study sites. This would have had the advantage of considering the DFs more coherently but would have made it more difficult to compare between study sites.

By giving the farmer a voice, we introduced subjectivity into the results. This could be a major limitation for certain research questions, and OHI data would need to be combined with other sources to get more balanced insight into the DFs from different perspectives (e.g. Bürgi et al. 2017; Berget et al. 2021). At the same time, this subjectivity can also be a strength, as the farmers’ rationale mirrors the realities they experienced. For example, a recent study found that local actors rather linked abandonment to fewer farmers, than to a decline in agricultural land (Dimopoulos & Kizos 2020).

Using a ‘rigid’ framework to analyse the OHI data inevitably led to some adjustments of the original DF framework, as real-world data often do not behave in a model-like manner. This is in line with Darnhofer et al. (2012), who state that farming systems are difficult to conceptualise because they integrate objective and subjective aspects. This is reflected in the difficulty the interviewees had making a clear—but ‘artificial’—distinction between external DFs and their own decision-making. For example, many farmers used expressions to both describe economic necessity and own motivation when explaining why they expanded their farms as the following quote illustrates: ‘So then we just continued on the 44 ha. We then realised that we had to grow [economically], because we did not want to stay farmers on that [small] farm in the future’ (Flevopolder (NL), F7). As such statements were considered essential, we decided to go beyond the usual definition of DFs as purely external and to include DFs for different types of ‘farm strategic planning’. In our experience, therefore, it is possible to use the DF framework to analyse (oral history) interviews, but the differences in the data also mean that some flexibility to adapt is required.

## Conclusion

The adoption of an actor-centred approach enabled insights into European farmers’ realities and factors that influenced individual farm change since the 1960s. Agricultural policy, although important, was mentioned as just one of several influential factors, highlighting the need to consider the whole diversity of potential DFs for understanding change, but also when designing interventions. Surprisingly, farmers did not perceive natural factors as essential DFs of change, most likely because they were taken as a given and as rather stable for a specific location—an aspect that may be changing under the increasing impacts of climate change. The long-term farmers’ perspective indicates high variability in DFs across localities, which cannot be fully captured by readily available, often large-scale data. Although there are recurring patterns of DFs across study sites, many important DFs turn out to be highly site-specific and linked to local context. In addressing European-scale challenges, these differences in the perception of DFs of the agricultural systems need to be acknowledged. Single DFs of long-term change are rare, DFs often operate in bundles, and interventions are likely to be most effective when addressing bundles of DFs. While we do not claim that the study sites considered here are exhaustively representative, we do cover important gradients that shape European agriculture (Diogo et al. 2023), and thus, the set of sites illustrates well the diversity of agricultural realities across Europe.

Including the perspective of farmers proved to be rewarding. To gain a more complete understanding of farm development it would be important to additionally include insights from other groups of stakeholders. By doing so, it would be possible to include different types of discourses and point out more precisely existing opportunities and barriers.

### Supplementary Information

Below is the link to the electronic supplementary material.

Supplementary file1 (PDF 2.87 MB)

Supplementary file2 (PDF 281 KB)

Supplementary file3 (PDF 127 KB)

Supplementary file4 (PDF 256 KB)

## Data Availability

The recorded and transcribed data of the oral history interviews cannot be shared due to privacy restrictions. The processed data are available from the corresponding author on request and subject to the rules and restrictions of the Swiss Federal Research Institute WSL.
