# Supplementary material for: Why has farming in Europe changed? A farmers’ perspective on the development since the 1960s
Source: Reg Environ Change. 2023 Nov 11;23(4):156. doi: 10.1007/s10113-023-02150-y (PMC10640510; doi:10.1007/s10113-023-02150-y)
Supplement: Supplementary file 1 — Supplementary file1 (PDF 2.87 MB) [file 10113_2023_2150_MOESM1_ESM.pdf]

Supplementary material: Why has farming in Europe changed? A farmers' perspective on the development since the 1960s (Mohr et al.)

Appendix I: Study site portraits

Contents

Tabular overview study sites 2
Study site portraits 5
St. Maria del Paramo (ES) 5
Colmenar Viejo (ES) 6
Lemnos (GR) 7
Lesvos (GR) 8
Querfurter Platte (DE) 9
Turzovka (SK) 10
Powiat Miechowski (PL) 11
Lielvircava (LV) 12
Ille-et-Vilaine (FR) 14
Scherpenzeel (NL) 15
Flevopolder (NL) 16
Reusstal (CH) 17
Hedmark (NO) 18
References 19

Tabular overview study sites

id	site name	country	High-impact political system[a]	max time span covered by interviews	farm types	dominating innovation	farm organisation	other income	land use
ch_reu	Reuss	Switzerland	-	1962-2020	livestock farms with dairy farms dominating; arable crop and grasslands	land melioration; milking machine/parlor	family farm and farm cooperation (successor: 8/10)	increase in parttime income, about half of the farms	Arable crops and grasslands
de_qup	Querfurter Platte	Germany	Socialist regime (1946-1990)	1960-2021	large scale arable crops; few mega stables	Increase in size of field machines / fields; advances in management of mega-stables; biogas plants	Kolkhozes / cooperatives; family farm (successor: 3/3)	today no other income mentioned; during socialist time individual interviewees farmed at home additonally to their work in the Kholkoze	Arable crops (dominating) and negligible, marginal grasslands
es_cov	Colmenar Viejo	Spain	Franco's regime (1939-1975)	1955-2021	dairy farming and meat production	milk machine/parlor; shearing machine;	family farm and part-time farm (successor: 2/9); 1 former cowboy for big landlord	some farmers were working fully off-farm	(dry) grasslands (dominating) and marginal arable crops
es_smp	St. Maria del Paramo	Spain	Franco's regime (1939-1975)	1971-2020	arable crops; singular sheep/dairy production	Irrigation system, precision seed drill; pesticide spreader	family farm (successor: 7/10)	individual farms	Arable crops (dominating) and some grasslands
fr_iev	Ille-et-Vilaine	France		1961-2014	dairy farming and meat production	milk machine/parlor; fences; cutting down of embarkments to enable mechanization	family farm (successor: 7/9)	individual farms;	Arable crops and grasslands

gr_lem	Lemnos	Greece	Military Junta (1967-1974)	1955-2021	sheep farming (for milk and meat)	new more productive breeds replace the local sturdier breeds	family farm (successor: 8/11)	halve of the farms had additional part-time income	Arable crops and grasslands
gr_les	Lesvos	Greece	Military Junta (1967-1974)	1957-2020	olive trees; some animals for own usage	roads/farm cars, olive nets, "shaker"	family farms (today mostly one-person undertakings) (successor: 3/10)	most had additional parttime/fulltime income	Olive groves
lv_lie	Lielvircava	Latvia	Part of Soviet union (1940/41; 44-1991)	1969-2021	arable crops; some livestock farms and orchards	Automatization of (harvest) labor through large machinery	Kolkhozes / cooperative; family farm (successor: 2/4)	individual, especially small farms, had other occasional/part time income	Arable crops (dominating) and some grasslands and orchards
nl_fle	Flevopolder	Netherlands	Creation of Flevopolder (1955-1986)	1969-2021	arable crops; some farmers are organic	Increasing complexity and size of field machines; weed burner and "treffler egg" for organic farms	family farm (successor: 7/10)	increase in part time/occasional income, about half of the farms	Arable crops (dominating) and some grassland
nl_sch	Scherpenzeel	Netherlands	-	1959-2007	livestock farms with very little arable farming	milk machine/parlor, temperature control for stables, egg packing machine	family farm (successor: 5/7)	no part time income common	Some arable crops and some grassland
no_hed	Hedmark	Norway	-	1955-2020	dairy farming and meat production; predominantly grasslands, some arable crops	'no-fence' technology; silage (both tower and baler); milk machine/parlor; stables that collected slurry	family farm and farm cooperation ("samdrift") (successor: 10/10)	individual farms	Grasslands (majority) and arable crops
pl_pom	Powiat Miechowski	Poland	Socialist regime (1946-1990)	1962-2019	mixed farm systems	More machinery (indet.)	family farm (successor: 10/10)	only one farm	Mixture of arable crops (majority), grasslands, vegetable / herbs, orchards

sk_tur	Turzovka	Slo-vakia	Socialist regime (1948-1989)	1974-2021	some large-scale livestock farms, most small "hobby" farms	More machinery (for mowing; indet.)	Kolkhoze / coopera-tive; part-time farm (successor: 2/5)	majority of farms worked full time elsewhere	Grasslands (domi-nating) and arable crops

[1]National political regime/event with high impact on land systems and agriculture during the time span covered by interviews

Study site portraits

St. Maria del Paramo (ES)

The study site is located north of the municipality of Santa María del Páramo and belongs to the autonomous community of Castilla y León, in north-western Spain. The agricultural district in which it is located is bordered to the east and west by the rivers Esla and Órbigo respectively, the confluence of these rivers marking its southern boundary, and the northern area is less well defined and is delimited by the foothills of the Cantabrian Mountain range. It has a continental Mediterranean climate and homogeneous topographical and climatic conditions, with a very gentle relief, extensive deforested areas and is situated at an altitude of approximately 810 metres. The current fluvial network is intensely transformed due to channelling and canalisations related to two concentrations of plots of land.

Until the beginning of the 20th century, the main crops were dry crops, e.g. barley, rye and wheat, as well as some vineyards, which were the only marketable crop, as the economy was basically subsistence. Essential livestock complemented the farms, which served as a workforce, as a source of organic fertilizer and also as a source of food. Water resources were very scarce, however, the lack of water was not total due to the existence of seasonal lakes and streams. During the first decades of the 20th century, productivity increased thanks to the construction of waterwheels and the improvement of the mouldboard plough, which allowed more agricultural land to be ploughed. This development led to a gradual drying up and loss of the lagoons as their livestock use was abandoned. At the same time, there was an increase in irrigated crops such as beans, potatoes and sugar beet. (Franco Pellitero, 1986; Domínguez Fernández, 2003; García Martínez, 2020).

During the 1960s, several events gave a definitive boost to the transformation from dry land to irrigated land: the construction of the Barrios de Luna reservoir, the development of the network of drains and irrigation channels and the implementation of the first land consolidation. Until the 1990s, crop diversity was high and rotational, with a form of irrigation mainly by flooding and where only a small area was irrigated by sprinkling, thanks to the annual assembly/disassembly of aerial pipes and the use of motor pumps. At the beginning of the 21st century, a new agricultural and landscape transformation began, which involved a second land consolidation and the modernisation of irrigation through the burying of the entire hydraulic network and the automation of irrigation, which began in the municipality under study in 2015. Since then, agricultural intensification has increased significantly, with maize as the main crop. Furthermore, during the last decades, family and extensive livestock farming has been gradually disappearing in favour of stabled and industrial livestock farms.

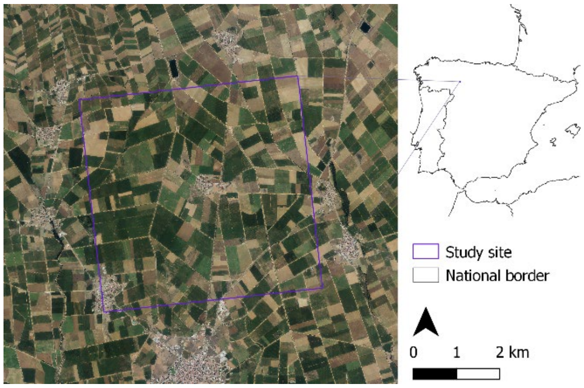

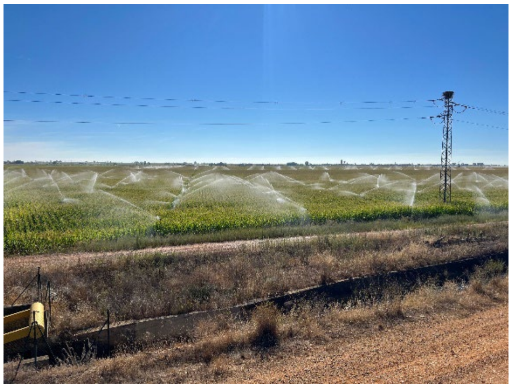

© Instituto Geográfico Nacional de España; Fabienne Frey

Colmenar Viejo (ES)

Colmenar Viejo is located between Madrid and the mountain range “Sierra de Gaudarrama”. Today the majority of land is covered by grasslands/pastures, part of which is wooded grassland (“Dehesa”).

Politically, Spain was under Franco's rule until 1975, which also affected agriculture, as the protectionist nature of the country led to price protection for agricultural products, but also hindered the exchange of innovation with other European countries. When Spain joined the EU (or rather its predecessor) in 1986, agricultural prices fell and hygiene standards, especially for milk, were introduced. During hygiene inspections, diseases such as brucellosis and tuberculosis were discovered on many farms, which subsequently led to widespread mass culling.

In the 20th century, an increase of settlement and a decrease of arable crops was observed throughout the municipality. Due to its proximity to Madrid and easy accessibility through a motorway built in the 1970s, the social fabric of Colmenar Viejo changed from an agricultural population to people commuting to Madrid or people from Madrid having a second home (Bürgi et al. 2017). During the same time part of the municipality was expropriated to create a new municipality called “Tres Cantos” specifically designed as a satellite city for the growing Madrid.

While flocks of sheep were common until the 1950s, they were replaced by dairy cows until the 1980s, when the trend shifted to beef cattle (Bürgi et al. 2017). Interviewees active in dairy farming reported how they could increase milk production back in the days thanks to mechanization but also how the massive killings of cows due to sanitary reasons, the low milk prices, and the lack of manpower made rearing cattle for meet more attractive (e.g. the Limousin breed) to some. Nowadays, most of them keep the farm as a secondary income and do not expect their descendants to take over the business.

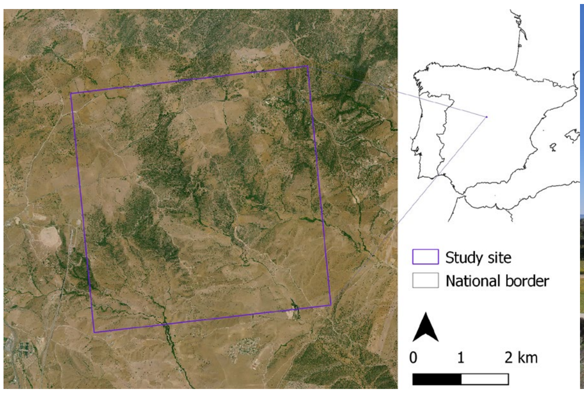

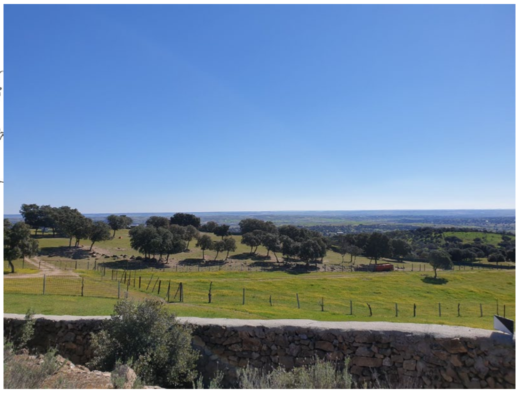

(c) Comunidad de Madrid; María García-Martín

Lemnos (GR)

The island of Lemnos covers 477 km². The island's climate is characterized as meso-Mediterranean with dry summers and mild winters (annual average temperature 16.1° C) and average rainfall at 467 mm/year. Major parts of the island have been designated as Natura 2000 areas (34,000 ha, GR4110001 and GR4110006), parts of which being agroecosystems. Compared to most Aegean islands, it is relatively level (highest point 430m a.s.l.), with more pronounced relief in its western and northern parts, dominated by dwarf-shrub vegetation (phrygana) and short grasslands, both being grazed by sheep and goats. In contrast, the flat and fertile central and eastern part is dominated by farmland mosaics, with livestock grazing around the rolling hills and arable land. Arable land is grown with cereals and legumes (for human or animal consumption) and many small vineyards are also found. Many farms are mixed (combining cultivation of crops and sheep/goat husbandry) and others exclusively oriented to arable farming (Georgiadis et al., 2022). With 3.7 stremmata (0.37 ha) the average patch size of cereal fields is very small, while it is much bigger for grazing lands, i.e. 68 stremmata (6.8 ha) per unit. The spatial configuration of these features leads to three landscape types: mosaic agriculture with limited presence of grazing lands; mixed (rangelands and arable fields); and rangelands.

Land ownership status has historically created a farm management system in which landowners provide the land and the "mandra" (farm house) and "kehagiades" (farmer/shepherd) provide labor and animals. Half of the farm's dairy and crop yearly products were given to the landowner as rent, whereas many other errands complete this complex relationship. As in other Aegean Islands (Spilanis and Kizos, 2016), the human population of Lemnos reached its peak in the early 1950s (exceeding 24,000 residents), declined until the 1980s (with a record low of approx. 15,700 in 1981), to slightly recover in the past 40 years (approximating 16,400 in 2021). More than 800 are full-time farmers (13.5% of the active population) and as many are part-time ones.

A crop disease in 1955 that affected the cultivation of cotton lead to a number of changes . It is considered a factor of out-migration and land abandonment as well as of the transition from cotton to cereals in the lowlands. Population decline also lead to structural changes in farm management as less working hands were available (Dimopoulos and Kizos, 2020; Bakalis, 2007). The introduction to the CAP mechanisms in the early 1980s marked a transition towards dependence to a degree from subsidies. These subsidies were originally handed out according to production data until the early 2000s, when they were linked to land use with the Single Farm Payment. CAP subsidies in Lemnos contributed to a rise in cereal production and the expansion of cultivation of crops to the edges of lowlands (Dimopoulos and Kizos, 2020).

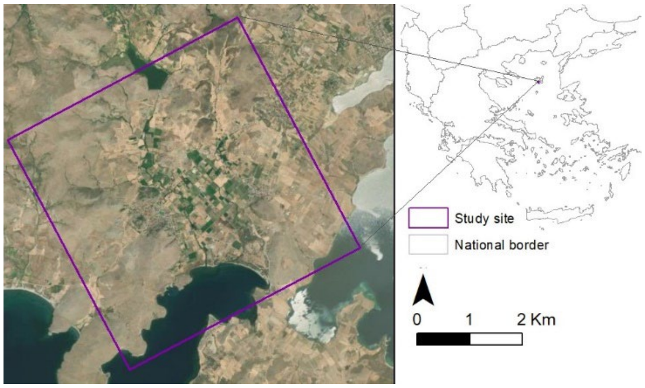

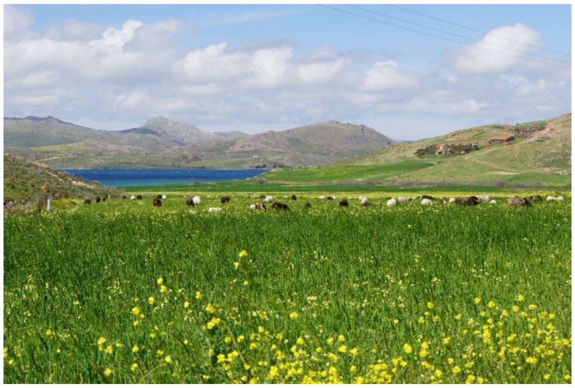

© Maxar satellite imagery basemaps; Stephan Meyer

Lesvos (GR)

Lesvos is an island located in the north-eastern part of the Aegean, covering an area of 1632.8 km2 and a population of 87,000. Olive cultivations are one of the two major land uses (roughly 35% of the total area), the other being grazing lands. Although the number of olive farmers has decreased in the last decades, still today roughly 16,000 farms are declared in the files of the Single Farm Payment of the Common Agricultural Policy. The landscape of the olive plantations is a homogenous landscape in the East and Southern parts of the island, terraced to a large extent which is characteristic for the island and part of the local identity (van der Sluis et al., 2014).

Olive plantations on Lesvos fall into the “traditional” and the “organic” categories, in the typology provided by the OLIVERO project (Stroosnijder et al., 2008 - the rest being “semi-intensive” and “intensive irrigated”), described by low labor and material inputs and manual harvesting (Duarte et al., 2008, de Graaf et al., 2008). The majority of the plantations fall into these two categories that are practically one category, since organic cultivation on Lesvos is very similar to “conventional” one. The inputs of this system (labor, capital, plant protection products and fertilizers) are low, as olives are harvested manually, with mechanical or manual clearing of the understorey and only harvesting requires high labor inputs. This system does not require high levels of mechanization or investments. Outputs (annual oil olive production) are generally low, as expected of this system, with biannual full harvests (called *maxouli* in the local dialect). The case study landscape (Gera) is located in the south-eastern part of the island. Its landscape is hilly and is dominated by terraced, continuous olive plantations (as high as 550 m a.s.l.), while only little other land is managed for other agricultural purposes. Some plantations have been abandoned or neglected in recent decades. Although the landscape is homogenous, the fields of each farm are small (around 0.4 ha on average) and each farm is dispersed in many fields, typically in distant locations (see Zagaria et al., 2018). According to official data (ELSTAT), Gera’s population has declined in the last decades (-37% from 1951 to 2011, and -13% between 2001 and 2011), in line with the trend for the whole island. The population is also aging, with more than a quarter of its population older than 65.

As for olive cultivation in general, the introduction to the CAP mechanisms in the early 1980s marked a transition towards dependence to a degree from subsidies. These subsidies were originally handed out according to production data until the early 2000s, when they were linked to land use with the Single Farm Payment. Due to the small farm size of many farms, the amounts received are not very big for most of the farms (the median was around 550€/farm according to Kizos et al., 2014).

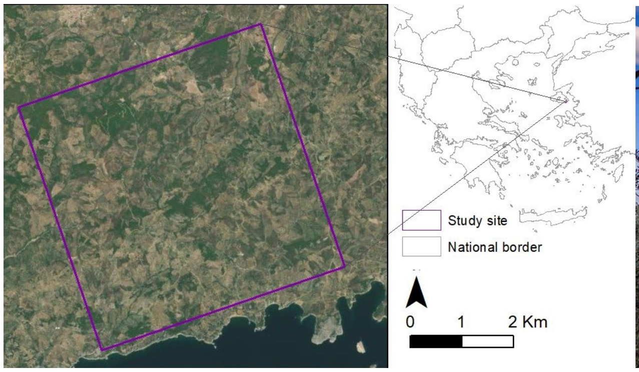

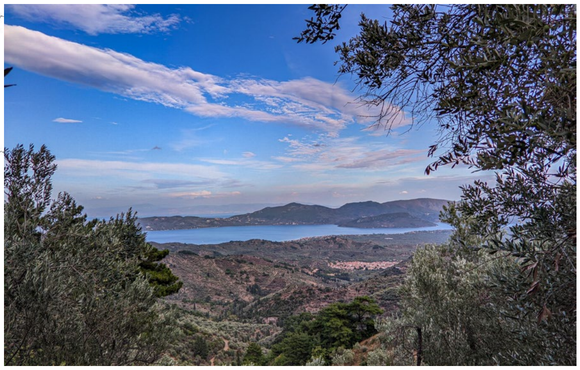

© Maxar satellite imagery basemaps; Stratis Sentas

Querfurter Platte (DE)

The Querfurter Platte is a fertile loess plain that is located west of Leipzig and south of Halle.

Through the socialist soil reformation in 1946, farms that were larger than 100 ha or belonged to former Nazis, were expropriated, and distributed among landless farmworkers, farmers with only little land, resettlers (mostly from former German areas like Silesia) and small land leasers. Soon after the SED encouraged farms to join and form collectivized farms, called “Landwirtschaftliche Produktionsgenossenschaft (LPG)”. Through increasing economic and juridical pressure, the full collectivization was reached by 1960. While in the beginning the LPGs were still relatively small and technologically low-key, new directives in the 70s to industrialize agriculture marked the start of the industrialisation of agriculture: This resulted in a state directed separation of plant and animal production, as well as a collapsing of the LPGs into larger institutions reaching up to 7000 ha for plant production LPGs. In the Querfurt district the Agrarindustrie Vereinigung Querfurt was created during that as a vessel to coordinate the work of a handful LPGs as well as adjunct holdings like the Agrar Chemisches Zentrum (ACZ) that took over fertilization, chemisation and melioration tasks for the LPGs and the organization of the collaboration with science. LPGs now often also had a social function, since they employed a lot of people from the villages and took over responsibilities, such as creating Kindergarten or building holiday homes for its employees.

Through the collapse of the socialist the farming system changed profoundly: LPGs were either dissolved or transformed into cooperatives. Families owning land, were allowed to lease them to whomever they wanted (if they were not cooperative members) or to start farming on their lands as “Wiedereinrichter” (re-settlers). At the same time Western Germans started to lease/buy farm land too, usually referred to as “Glücksritter” (fortune knights) by the interviewed farmers. To soften the blow of entering the capitalist system, certain tools were put into place, like the possibility for early retirement. In recent years CAP regulations had some impacts with regard.

After the unification, a lot of infrastructure was built in the area, like the IC line or a highway, leading to a loss of agricultural land for some of the farmers. In recent years, wind mills as well as biogas plants, partly built also by interviewed farmers/cooperatives, started to shape the landscape. The interviewed farmers that were “Wiedereinrichter” had large scale arable farms, while the interviewed cooperatives kept on both an animal production branch as well as large-scale areas of arable crops after the Wende.

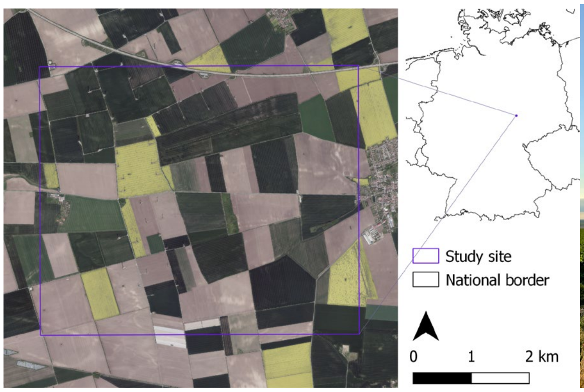

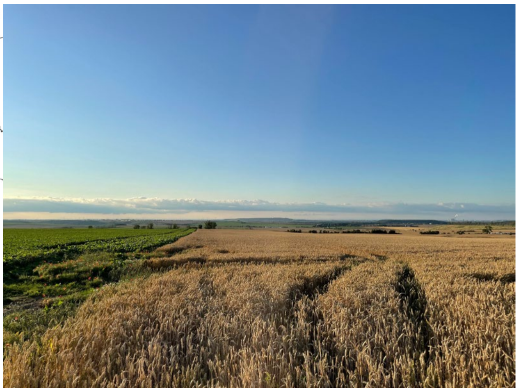

© Geodatenportal Sachsen; Franziska Mohr

Turzovka (SK)

The study area is located in the northwestern part of Slovakia at the border with Poland and the Czech Republic. The area is close to these countries' frequent road and railway nodes of international transportation networks. The landscape especially housing is characterized by a mix of traditional and so-called “socialistic” architecture. The scattered settlement and small parcels on higher slopes, however, creates one of the most significant regions of traditional agricultural landscapes in Slovakia (Izakovičová et al., 2022). Socialism, related industrialisation, collectivisation of the agricultural land and private business, subsequent postsocialism and accession to the EU largely changed the demography and landscape structures. From the demographic perspective the political changes brought turbulent times of population migration (www.turzovka.sk). From the agricultural perspective, the most favorable agricultural land containing (arable land and mostly grasslands) in the valle bottom was collectivized during socialism, resulting in the reduction of the number of agricultural plots, increased plot area sizes and increased number of livestock production (www.turzovka.sk). In the less favorable areas, mostly during socialism, local farming was kept active as a free-time activity of the workers commuting to their industrial and administrative jobs in the neighborhood. In the late 1980s and till 1990s, most of the (relatively large) houses' backyard gardens were largely used for hobby agricultural and livestock production (as noted in the interviews “almost every house owner kept one or few cows during the socialism”). In the late 1980s and further, the characteristic scattered settlement in the surrounding hilly areas turned continuously to the secondary houses. The ownership of these scattered settlements changed to locals that lived in the nearby cities, but also to the people with permanent residence in distant cities (without real-estate agencies, the secondary housing was grounded on references which often turned out that enclaves from certain cities occupied the same clusters of the scattered settlement.

Hobby farming largely disappeared in the last 30 years due to generational exchange and also due to the out-migration of the population. With the hobby farming also disappeared the croplands as nowadays only managed grasslands represent the agricultural plots. The out-migration phenomena became typical for many of the rural areas in Slovakia and was even boosted by the accession to the EU and Schengen Area. Nowadays, the surrounding landscape is managed mainly by agricultural enterprises farming on mostly rented parcels as the ownership is largely heterogeneous due to the complicated restitutions. However, exceptionally to the other such valleys in Slovakia, also few small-scale farmers may be found here that keep diverse farming practices and livestock production. Nevertheless many of the parcels on higher slopes are abandoned with presence of mature forest structures.

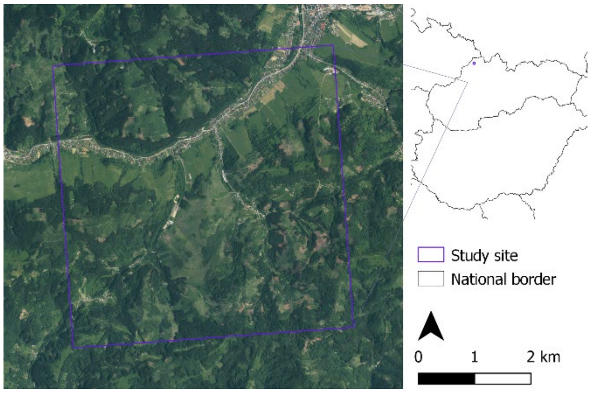

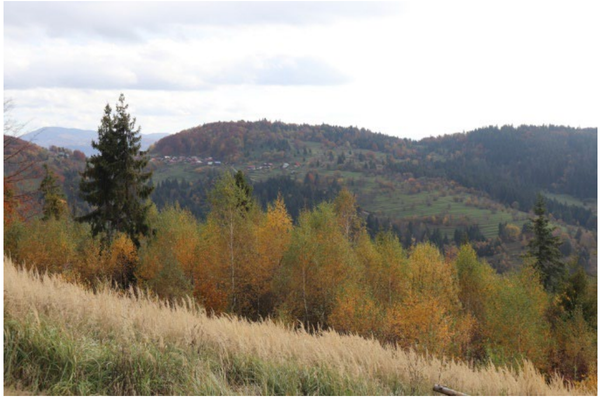

© Geoportal.sk; Robert Pazur

Powiat Miechowski (PL)

The study area is located in the Miechowski District, which belongs to the Małopolskie Voivodeship, in the South of Poland. The district is situated mainly located in the Miechowska Upland, whose fertile soils of support biodiversity. The upland landscape consists of variable bedrock underlying the soils, varied slopes and a dense network of field boundaries and ravines on loess slopes. Miechowski district has nine nature reserves, some of which are steppe, forest and floral types. Twenty-one Natura 2000 Special Areas of Conservation have been established to protect specific types of natural habitats and species considered valuable or threatened in Europe, such as Carlina acanthifolia subsp. utzka (Hacq.) Meusel & Kästner and Cypripedium calceolus.

After the Second World War, the Polish socio-political system changed. There are two main periods of change in Polish farms and landscape:

(1) From 1945 to 1989 (socialism): The agrarian reform followed the socialist pattern and included the nationalisation of large holdings and processing plants. The attempt to eliminate individual holdings by forced collectivization (farmers had to join agricultural production collectives) failed. Individually owned holdings survived and amounted to approx. 70% of all agricultural holdings. Despite mandatory quotas of agricultural products, shortages of goods—even basic ones—were commonplace. Available items usually were rationed and one needed ration coupons to buy them. On 13 December 1981, martial law was imposed in an attempt to crush the democratic opposition and defend the communist regime. Although martial law was formally lifted on 22 July 1983, meat ration coupons were used until 1 August 1989. The rationing was a countermeasure for significant shortages at the time.

(2) after 1989 (the year of the socio-political transformation in Poland): Socialist restrictions on agriculture were lifted, such as mandatory quotas or obligatory production from all pieces of agricultural land. Recent decades have seen a significant oversupply of agricultural products, aggravated financial problems for farmers, and a decline in agricultural land (mostly converted to forest and developed land (Bański, 2016)). Poland's membership of the EU has also brought with it necessary and daunting reforms of the Polish agricultural industry and subsidy system (costly for both farmers and the public).

Today, Miechowski District is one of the largest manufacturers of open-field vegetables in Małopolskie Voivodeship. Vegetable cultivation is a promising branch of agriculture in the Miechów area. The municipalities in Miechowski District are famous for their agricultural products: Charsznica for various cabbage cultivars (called the cabbage capital of Poland), Słaboszów for its milk and sugar beets, and Książ Wielki for pig farming and meat packing. However, a large portion of equipment and machinery is obsolete and in poor condition. The upgrade process takes very long because of low farming profitability and high machinery prices. With very good soils and favourable environmental conditions (no industry), farming is the strength of the district. The average holding in Miechowski District is 8.09 ha (Agricultural Census 2020). Farmers from Miechowski District receive subsidies, such as direct subsidies, or subsidies for areas with natural limitations (unfavourable conditions). Other numerous efforts are made to support farmers, such as regarding animal well-being. Other grants include payments for beekeepers, afforestation schemes, fertiliser subsidies, and pig herd size maintenance. Most of the residents work in agriculture. Therefore, the region's future is generally associated with organic farming, agricultural and food services, and agritourism. The entrepreneurial spirit of the local population led to the district winning the 'Golden Euro" prize in 2005 for the largest amount of external funds in Małopolskie Voivodeship in the first year after Poland joined the EU.

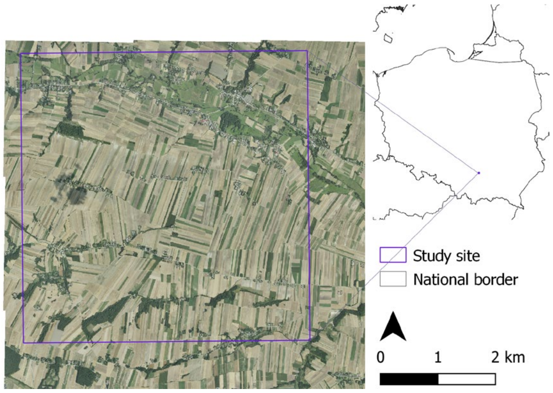

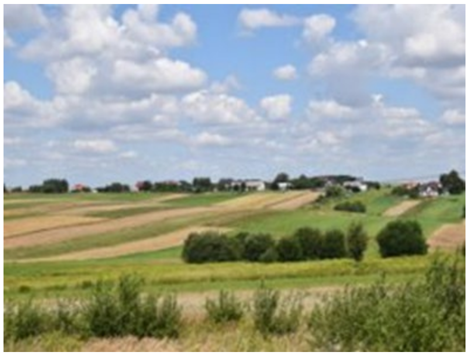

© Józef Hernik

Lielvircava (LV)

Lielvircava is located in the central part of Latvia (administratively, Jelgava Region, Platone Parish), in the midst of the fertile Zemgale Lowland and represents the Latvian iconic landscape of intensive agriculture. The region is formed from clay sediments of a glacial lake, which in the context of Zemgale often are called "the fatty clay" creating historically important preconditions for intensive agriculture (e.g., in 1935 croplands comprised more than 80% of agricultural land). Climate is mild and humid with precipitation surplus in July and August (the average annual air temperature is +5.9°C, the average annual precipitation – 667 mm). The spatial structure of the flat lowland is determined by a dense river system which forms the basis of historical settlement structure – the distribution of farmsteads, manors and roads. This peculiar landscape structure has changed due to the 20th century land reforms, creating a more even distribution of settlements – single farmsteads and villages (population density in Platone Parish in 2022 is 16/km^2). The inherently linear spatial structure of today's landscape manifests itself also in various man-made regular shapes – lines of windthrown trees, straight roads, straightened small rivers that are mostly a result of landscape adaptation for a large-scale agro-industrial production.

The landscape of Lielvircava has been impacted by several political regimes during the 20th century: manorial period with large estates and peasant farmstead settlements (Lielvircava manor was one the biggest in the region, appr. 16 thousand ha); interwar period with nation state's land reform, nationalization of manorial lands and assigning it to the so-called new-farmers (with average size of 20-30 ha); Soviet regime with collectivization of land and establishing of kolkhozes (in the 1980s, the kolkhoz Red Star in Lielvircava was one of the smallest but also among the wealthiest in Latvia with appr. 1200 ha of agricultural land); restitution of land to its previous owners and transitions to capitalist market-economy in the independent Latvia since 1990. In the Lielvircava case, the kolkhoz was transformed into a shareholding cooperative, succeeding into a limited liability company now managing altogether appr. 2500 ha of agricultural land (both owned and leased from private owners).

The agricultural landscape in and around the study area can be characterized as highly intensive with large fields predominantly of winter wheat and winter rapeseed. Dairy farming has declined overall in the region, a few niche farms are developing (mainly fruit orchards and vegetable farms). Since the beginning of 2000s there has been a considerable decline in farm numbers. In 2006, 104 farms in Platone Parish were receiving area payments, in 2019 – 61. Based on the interviews and the agricultural regime analysis, two time periods in the 20th century, decisive for today's landscape and farming character, stand out. First is around the end of 1970s and through 1980s when structural changes in kolkhozes took place along with the mass amelioration and massivization of fields to boost

agricultural productivity (Melluma 1994). In Lielvircava's kolkhoz Red Star fruit orchards were liquidated (app. 70 ha), agricultural produce diversity reduced (e.g., small poultry farms) focusing on dairy farming, and more commercial crops, such as sugar beets. The other important period is the 1990s marked by the transition to capitalist market economy. Land restitution and the possibility to buy agricultural land created a wave of new farming activities, which eventually shrunk due to the times of economic uncertainties of the 1990s. According to the interviews, the lack of State's support, market instabilities, lack of machinery and farming knowledge were the main factors to give up farming, with only few succeeding in agri-business. The main local narrative, to which many interviewees agree upon, is that only the big farmers (with arable land over 500 ha hectares) and niche farmers can thrive in this fertile agricultural region.

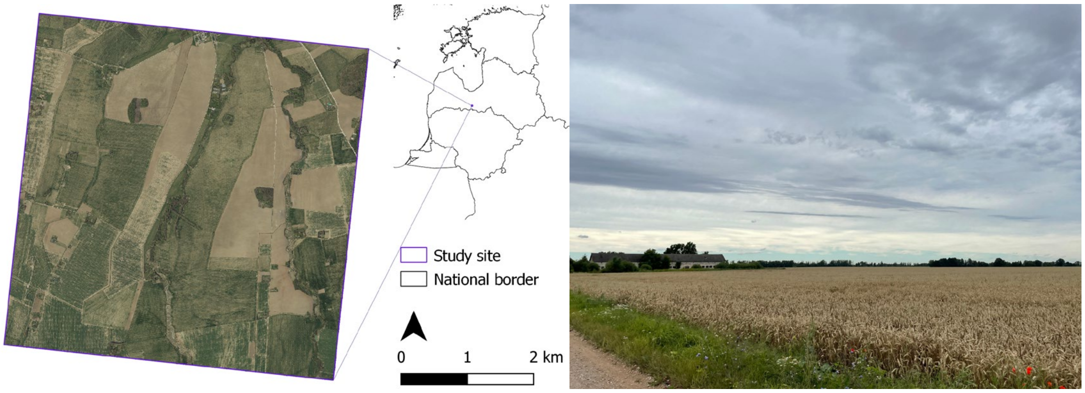

(c) Dodies.lv; Franziska Mohr

Ille-et-Vilaine (FR)

The study site is located in north-eastern Brittany, in Ille-et-Vilaine « département » (NUTS3). The study area has an altitude between approximately 75 and 110 m.a.s.l. and is characterised by a gentle hilly landscape. Granitic bedrock, interspersed with sedimentary material, dominates in the geological subsoil in the area where farmers' interviews were made. The climate was considered as temperate – oceanic. The characteristic feature of these agricultural landscapes is the "bocage structure", in this case hedgerows traditionally made up of rows of pruned trees on embankments surrounding the fields (notably used to mark property boundaries and to produce firewood and timber in the 19th and early 20th century, in a context of diversified agriculture).

After the Second World War, the Breton agriculture adopted a strong modernization mindset, in line with changes at French and European level, but also to make up for what was seen as a development lag compared to other regions. An essential pillar of the development described was the importance of the formal and informal social networks of the rural population (e.g. trade unions, cooperative organizations –for trading, for agricultural equipment use-, Christian youth associations, agricultural extension groups). All these organizations played an important role in disseminating knowledge and new technologies and contributed to the development of the "Breton agricultural model". This study site was shaped by the development of specialized dairy farming systems based on fodder maize and temporary grassland, for long distribution channels (noticeable development of dedicated manufacturing industries in Ille-et-Vilaine). In this context of agricultural development and fragile surface water resources, water pollution by organic effluents and pesticides has increased sharply. In the area, land-consolidation programs at municipality level have not been decisive instruments for farm expansion, unlike in other part of Brittany. With a widespread mechanization and industrialization of agriculture in this period, many of these bocage structures were removed to increase the size of fields and make them more accessible.

The 1980s marked a clear break with the introduction of milk quotas as a European policy measure, against a background of ever-increasing milk surpluses. Although some diversification has taken place, overall dairy specialization has been maintained, with a sharp reduction in the number of farms and a sharp increase in their size. The economic evolution accompanied by a further expansion in farm size, with a stable trend in the number of workers per farm, has led to a growing degree of mechanization and motorization and more recently to an increase of the area with annual cash crops. Individual and inter-individual land reorganization processes have taken place, when new plots have been absorbed by the remaining farms, leading to further plot enlargement and hedgerow removal. A slight slowdown in the erosion of the bocage has been observed in the study site since the turn of the millennium: trees are being maintained to a greater extent in areas that are more constraining for agriculture (e.g. valley bottoms), and some new multi-species hedgerows have been planted with the support of dedicated policies.

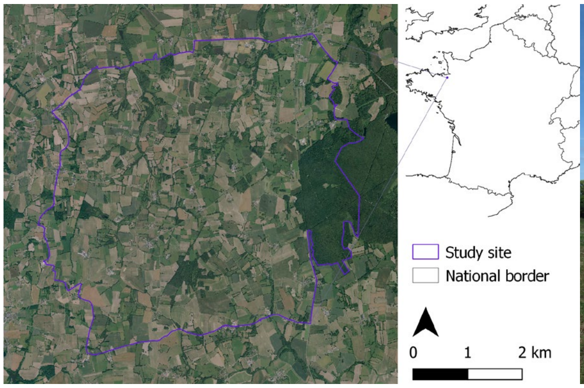

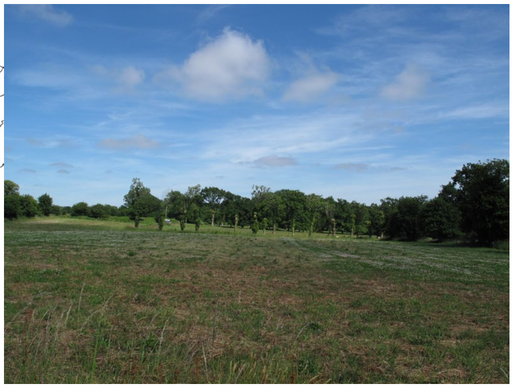

© IGN (2021); Leo Müller

Scherpenzeel (NL)

This study site is located in the southern, lower part the Gelderse Vallei. As this area had humid soil, it had to be drained to become suitable for agriculture. Through the process of drainage, a so call “Kampenlandschap” consisting of a small scale, mosaic shaped landscape pattern with farmland fenced by wooded banks was created. In the 19th and 20th century the landscape was then optimized for agricultural use by a normalization of streams, land consolidation and the removal of plantations around fields (Brons & Groot-Koerkamp 2005). Although historically the land was used for mixed farming with considerable portions of arable land, most of the land is sandy soils that have limited production capacities and makes thus the use as grasslands or for corn production the better option (Province of Utrecht, 2015; Werkgroep Snijmais 2020). Consequently, nowadays, the focus of the majority of farms is on livestock farming (CBS 2009). While agriculture plays still an important economic role in this region, in the past decades housing expanded considerably and led to a loss of agricultural land and the moving of farms

After the 2nd World War, the Dutch government designed an active modernization policy to promote a good living standard for farmers, low consumer prices and an increase of export of agricultural products. When wages in other sectors increased, the Minister of Agriculture introduced a ‘Meerjarenplan voor ruilverkaveling’ (long-term programme for land consolidation) in 1958 to increase labor productivity by enhancing farm enlargement and exchanging parcels between farmers to allow larger consolidated fields and farms. This policy implied the creation of an infrastructure that enabled farms to produce more intensively. During this time also the number of tractors, combine harvesters and milk machines increased, partly thanks to money from the Marshall Fund (Karel, 2017, Bieleman 2010). When in 1961 one the Common Agricultural Policy (CAP) of the EU was created, fixed prices led to an incentive to increase production, which then tilted over into an overproduction (more than the demand) (Brakel 2020). To limit the production quantities, first milk quotas were introduced on a EU level and then the CAP changed with the MacSharry reform from production support to direct payments in 1992. From 2003 onward the CAP cut the link between production and subsidies entirely and asked for more environmental, (food) safety, health and welfare standards to be met to be eligible for income support (Oenema 2013, European Commission 2020). On a national level, most farms in 1950 were still small extensive mixed farms (1950: 410’000 registered, mean size = 5.7 ha), whereas today the farms increased in size and specialized (2016: 55’000 registered farms, mean size = 32.4 ha) (CBS, 2017). A similar trend was observed for the Scherpenzeel farmers with all of them starting out as mixed farmers that then specialized on cattle, dairy cow, chicken, or pig farmers later in their careers.

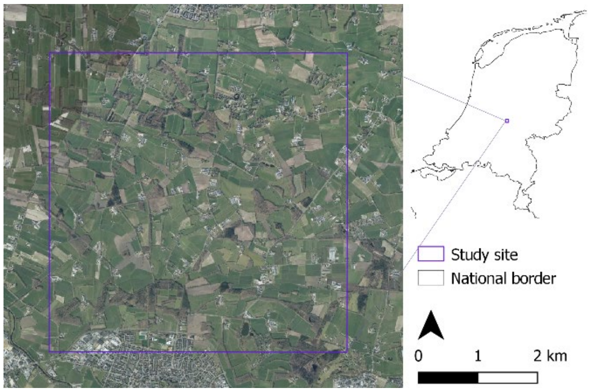

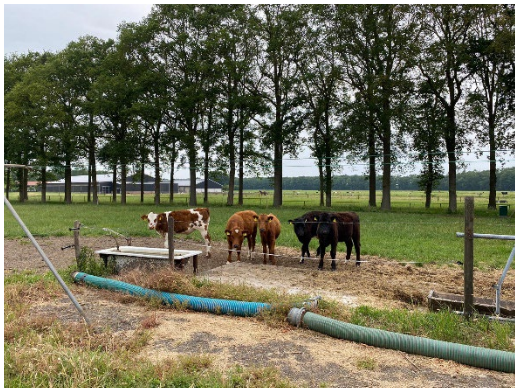

© PDOK; Rebecca Swart

Flevopolder (NL)

This study site is located in the city of Dronten, which is situated in the north-eastern part of Flevoland. Flevoland has only a very recent history: it is a polder that was created between the 1950s and 1960s as part of the "Zuiderzeewerken" project. This project was realized by the Dutch government from 1930 onwards to cope with the vulnerability of the Dutch food supply perceived during the First World War and the increasing population pressure. Because the Flevopolder was created from scratch, plot sizes were determined in a way that was ideal for large-scale and efficient farming operations without having to take into account pre-existing landscape features (Haartsen, 2018). To populate the newly gained land, farmers from the mainland could apply for a farm in the Flevopolder. Selection of farmers was based on skills, motivation and ensuring a representative mix of origins and religious background. Successful applicants got a leasehold that could later be bought off the government. In Flevoland, about 70% of the agricultural land is used for arable farming and 21.1% for grass and green fodder production. About 50% of the farms are arable farms, the rest is a mix of livestock, horticulture, permanent and mixed farms (Vogelzang et al., 2019). Flevoland is not only known for its high productivity, but also for a high number of organic farms compared to the rest of the Netherlands. In recent years, the city of Dronten expanded and grew closer to the agricultural area.

In the interviews, a few interesting observations regarding the farm structure and strategies of the farms interviewed were made: about half of the farmers were still in a partnership with their sons (-in-law) to enable the offspring to enter the farm and to ensure a smooth transition. Also, it was interesting to note the high economic importance of quality certificates from international companies such as GlobalGAP, even though such programs come with substantial additional costs, high bureaucratic effort, and extra controls. Almost all farmer families generated some additional income outside their own farm holding.

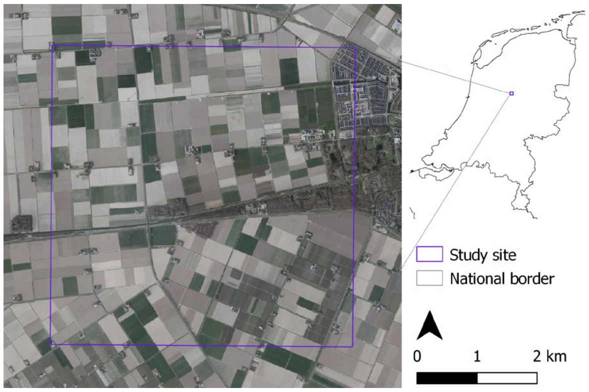

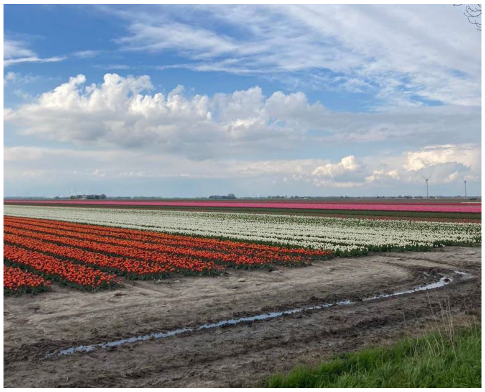

©PDOK; Nikki Odenhoven

Reusstal (CH)

This study site is located in the Reuss plain and intersects the municipalities of Mühlau, Merenschwand, Aristau, Rottenschwil and used to consist of small agricultural fields and meadow orchards. In the 70s/80s, there was an extensive land consolidation, which allowed farmers to better arrange the agricultural fields and thus increase the size of them, as well as increased production of arable crops thanks to accompanying land melioration with new drainages and soil improvement. However, at the same time, areas were put under nature conservation in the sense of land sharing - land sparing. This created a leisure area for residents, while also creating side jobs for a few farmers. On the other hand, the land consolidation and melioration process were very time and cost-intensive, which led to a decrease in the number of farms.

The overall political situation in Switzerland was stable during the study period. Related to that is the introduction / abolition of the milk quota that impacted farmers strategies. However, with the changeover of agricultural policy in the early 1990s from market support and product guarantees to direct payments, which are closely linked to minimum ecological and animal welfare standards, the institutional framework conditions for farming systems were fundamentally redesigned. New regional policies also allow farmers to increase their income through the participation in additional voluntary ecological programs / biodiversity schemes. Despite the subsidies, many farmers gave up their farms in the 1990s due to the new requirements imposed by the new agricultural policy."

On a regional level, the already mentioned land consolidation was a major political change that had an influence on the field size, as well as on the further growth in farm size with a simultaneous decline in the number of farms. During the study period, all municipalities experienced population growth as well as increased real estate development due to the attractiveness of the municipalities for commuters given their proximity to major urban centers.

Already in the beginning of the 20th century, this region has been described as specialized in dairy farming. Today, most farms are still oriented towards animal husbandry, with a primary focus on dairy production, but also suckler cows and pig farming. Due to the location of his farm in the village, one interviewee was not able to develop his farm as planned, leading to an unusual specialization in Christmas trees. Another interviewee joined forces together with another farm in order to increase in scale.

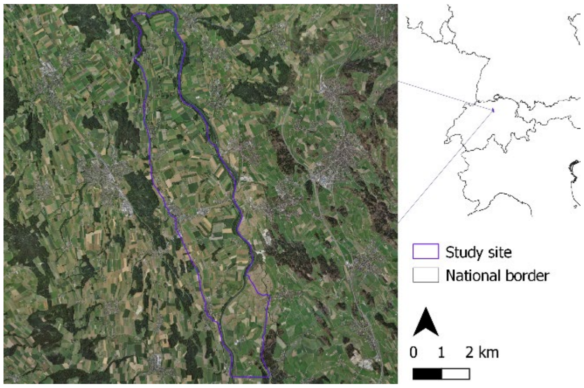

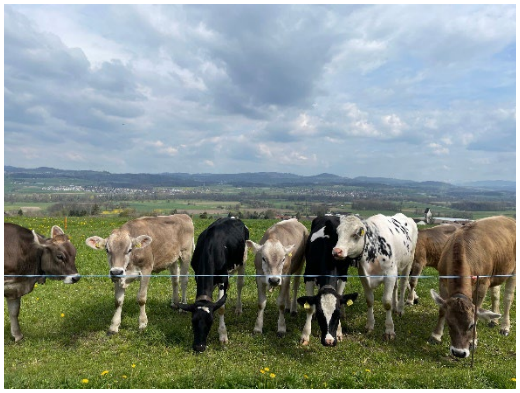

© swisstopo; Livia Lehmann

Hedmark (NO)

This study site is located in Vingelen, a small village with about 500 residents in the municipality of Tolga, located in «Østerdalen» in the northern part of the county «Innlandet». The altitude of the village is approximately 700 m.a.s.l. In general, the farms are situated in the middle boreal vegetation zone (valleys) and the northern boreal zone, and many farms have parts of their land in both zones. The climate is characterized by cold winters and a relatively warm, but short summer season. The soils in the area are fertile, but fields tend to be on the slopes of which some can be fairly steep.

The development of the farming structure in Vingelen has in many ways followed a similar trend as the rest of Norway and Europe with effectivization and industrialization. In the 1950's, the village had many small and diversified farms. Outfield resources were used extensively, both for winter fodder and grazing and most farmers took their herds to mountain shielings during summer. There were also local dairies and slaughterhouses. Most farms had several different kinds of animals, but dairy production and sheep farming were dominating. Farms produced for sale, but also for the household. Industrialization and effectivization resulted in a reduced number of farms that manage larger herds and more land.

While most of the agricultural land is still cultivated, the number of farms has decreased dramatically and the farms are mostly specialized, either dairy farms, sheep farms or with suckler cow / meat production. The current farmers often rent land from other landowners that are no longer themselves farming, or they have purchased land from neighbours and relatives.

In 1975 the so called «Opptrappingsvedtak» was decided by the Norwegian government. This involved increased subsidies and a goal to increase the income of farmers, making it comparable to the average income of industry workers. From 1975 to 1985 farmers received significant subsidies for cultivation of new land. In Vingelen this resulted in extensive cultivation of land, much of it at higher altitudes than the existing farmland. Some farms doubled or even tripled their amount of land in this period. Number of animals increased accordingly.

Due to increased subsidies, increasing area of farmland and number of animals during this period, farmers also invested in new barns and other buildings. From the 1990s the subsidies have again decreased and international trade agreements and increased international competition have caused new challenges for profitable food production, especially in areas with challenging growing conditions, like Vingelen. Based on various agricultural policy aims (e.g. self-sufficiency), the government subsidies favour farms in these districts. Still, most farmer households in Vingelen today derive most of their income from other lines of work.

Another national political event that influenced agriculture in Vingelen was the introduction of milk quotas in 1983. The goal was to curb overproduction, but the distribution of quotas among farmers did not always give the best distribution from a local perspective. According to the interviews, also the cessation of local dairies and slaughterhouses have influenced farmers in the Village.

Vingelen is characterized by a high level of social cohesion. This has enabled cooperation among farmers when it comes to equipment, fencing in outfield areas and more and is an important reason for the continuation of active farming and use of outfield resources in the area.

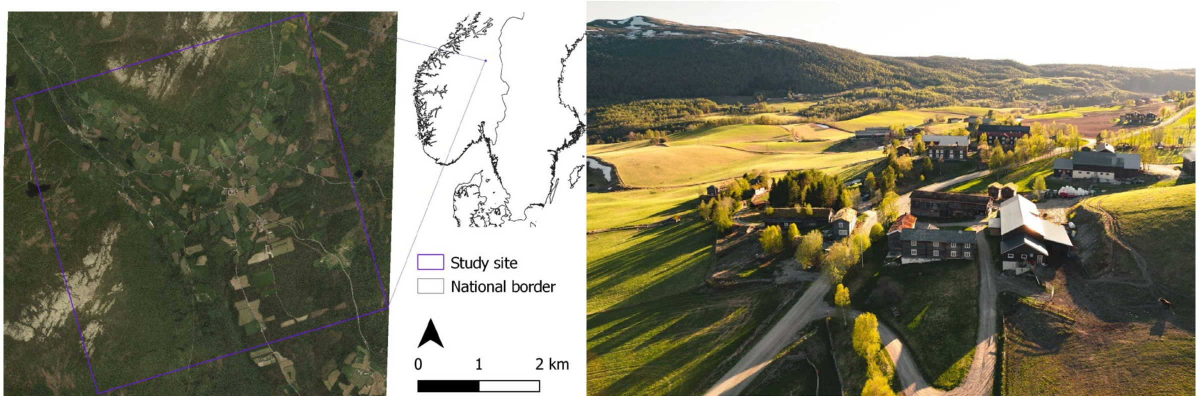

© Norge digital; https://vingelen.com/ (accessed June 2023)

References

Santa Maria del Paramo (ES)

Domínguez Fernández R (2003) Los regadíos del Páramo Leonés y su repercusión en las zonas húmedas. In M. Drain Mothré (ed) Politiques de l'eau en milieu méditerranéen. Le cas de la péninsule Ibérique Alicante: Universitat d´Alacant / Universidad de Alicante, Casa de Velázquez, Alicante, pp. 113-124)

Franco Pellitero D (1986) Transformaciones del espacio agrario en el Páramo de León. León: Diputación de León, Institución Fray Bernardino de Sahagún

García Martínez J (2020) El Páramo Leonés y los recursos hídricos: transformaciones en época contemporánea. Trabajo Fin de Grado. Universidad de León

Comenar Viejo (ES)

Bürgi M, Bieling C, von Hackwitz K, Kizos T, Lieskovský J, et al (2017) Processes and driving forces in changing cultural landscapes across Europe. Landsc Ecol 32:2097–2112. https://doi.org/10.1007/s10980-017-0513-z

Lemnos (GR)

Bakalis C (2007) "Lemnos: Urban structure, social restructuring, migratory networks and urban reflections in the 19th and 20th century", Doctoral Thesis, University of Aegean, School of Social Sciences, Department of Sociology.

Dimopoulos T, Kizos T (2020). Mapping change in the agricultural landscape of Lemnos. Landscape and Urban Planning. 203:103894 https://doi.org/10.1016/j.landurbplan.2020.103894.

Georgiadis NM, Dimitropoulos G, Avanidou K, Bebeli P, Bergmeier E, et al: (2022) Farming practices and biodiversity: Evidence from a Mediterranean semi-extensive system on the island of Lemnos (North Aegean, Greece), Journal of Environmental Management, Volume 303, 1 February 2022, 114131, https://doi.org/10.1016/j.jenvman.2021.114131

Lesvos (GR)

Stroosnijder L., Mansinho MI, Palese AM (2008) OLIVERO: The project analysing the future of olive production systems on sloping land in the Mediterranean basin, Journal of Environmental Management, 89:2:75-85, https://doi.org/10.1016/j.jenvman.2007.05.025.

Zagaria C, Schulp CJE, Kizos T, Verburg PH (2018) Transformation and persistence in Mediterranean landscapes: A case exploration of agricultural abandonment integrating farmer decision-making and tourist landscape preferences in east Lesvos, Greece. Regional Environmental Change. https://doi.org/10.1007/s10113-017-1276-4

Kizos T, Kolovos C, Metaxakis M (2014) Choriki diaforopoiisi ton dikaiomaton eniaias enisxisis tis KAP stis Perifereiakes Enotites tis Elladas [Spatial differences of Single Farm Payments in Regional Units of Greece], Geographies, 24: 91-106 (In Greek).

Turzovka (SK)

Mesto Turzvoka (accessed June 2023) www.turzovka.sk

Izakovičová Z, Špulerová J, Raniak A, 2022. The Development of the Slovak Agricultural Landscape in a Changing World. Front. Sustain. Food Syst. 6:862451. https://doi.org/10.3389/fsufs.2022.862451

Powiat Miechowski (PL)

Bański J (ed) (2016), Atlas obszarów wiejskich w Polsce. IGiPZ PAN, Warszawa.

Agricultural Census 2020. Central Statistical Office, Warsaw.

Powiat Miechowski (accessed June 2023) https://www.miechow.pl

Lielvircava LV

Melluma A (1994) Metamorphoses of Latvian landscapes during fifty years of Soviet rule. *GeoJournal*, *33*(1), 55-62

Scherpenzeel (NL)

Bieleman (2010) Five centuries of farming. Wageningen: Wageningen Academic Publishers.

Brakel A (2020, Juni 15) *Van melkbussen tot boterbergen*. Retrieved from Historisch nieuwsblad: https://www.historischnieuwsblad.nl/van-melkbussen-tot-boterbergen/

Brons & Groot-Koerkamp (2005) Landschapsontwikkelingsplan Gelderse Vallei. Culemborg: Brons Partners.

CBS (2009, March 30) Landbouw; gemeente, 1980 - 2000. Retrieved from CBS StatLine: https://opendata.cbs.nl/statline/#/CBS/nl/dataset/7316SLLB/table?dl=36BF1

CBS (2017, January 31) Agricultural production in the period 1950-2015. Retrieved from CBS: https://www.cbs.nl/en-gb/news/2017/05/agricultural-production-in-the-period-1950-2015

European Commission. (2020, March). Aims of the common agricultural policy. Retrieved from European Commission:https://ec.europa.eu/info/food-farming-fisheries/key-policies/common-agricultural-policy/cap-glance_en#Timeline

Karel E (2017) *Essays over effecten van de modernisering van het boerenbestaan in Nederland (1945-2012).* Groningen: Historia Agriculturea.

Oenema (2013) Transitions in nutrient management on commercial pilot farms in The Netherlands. Wageningen: Wageningen University.

Province of Utrecht (2015, February 12) *Factsheet 20. Gelderse Vallei : zand - zeer droog tot vochtig* . Retrieved from GEO Provincie Utrecht: https://geo.provincie-utrecht.nl/publiek/documenten/bodem/bodemsysteemwijzer/factsheets/20.pdf

Werkgroep Snijmais - Wageningen Livestock Research en Wageningen Plant Research (2020) *Handboek snijmais.* Wageningen Livestock Research en Wageningen Plant Research, Wageningen

Flevopolder (NL)

Haartsen T, Thissen F (2018) Physical and Social Engineering in the Dutch Polders. Physical and Social Engineering in the Dutch Polders: The Case of the Noordoostpolder. In Jones R & Diniz AMA (Ed) Twentieth Century Land Settlement Schemes. CRC Press, Boca Raton, pp.159-178. https://doi.org/10.4324/9781315167886-9

Vogelenzang TA, Smit AB, Kuiper PP, Gillet C (2019) Grond in beweging; Ontwikkelingen in het grondgebruik in de provincie Flevoland in de periode tot 2025 en 2040. Wageningen Economic Research 2019:003. https://doi.org/10.18174/464860o
