# Supplementary material for: Why has farming in Europe changed? A farmers’ perspective on the development since the 1960s
Source: Reg Environ Change. 2023 Nov 11;23(4):156. doi: 10.1007/s10113-023-02150-y (PMC10640510; doi:10.1007/s10113-023-02150-y)
Supplement: Supplementary file 2 — Supplementary file2 (PDF 281 KB) [file 10113_2023_2150_MOESM2_ESM.pdf]

Supplementary material: Why has farming in Europe changed? A farmers' perspective on the development since the 1960s (Mohr et al.)

Appendix II: OHI questionnaire

Past land use development: Informed consent form

Today's agriculture is under enormous pressure. On the one hand, productivity should increase in order to feed the growing world population; on the other hand, cultivation should become more sustainable. The SIPATH project of the Swiss Federal Institute for Forest, Snow and Landscape Research WSL (Switzerland), Agroscope (Switzerland), and the Free University of Amsterdam (The Netherlands) investigates how these two needs can be combined in the present and future agriculture.

One part of the project focuses on past land use developments in order to better understand what influenced changes in land management. For this, we study how farming and the life in agriculture has changed in 14 different landscape in Europe. Together with [*replace with institution of study partner*], we conduct in [*replace with study site*] up to 10 oral history interviews with former farmers and farm workers.

In the interview, we ask questions about how life on the farm, farm organization and land use intensity have changed since you started to work in agriculture. The answers you provide will help us to understand the past development in agriculture, as well as the challenges and reasons for decisions on the farm.

There are no risks involved in participating in this study. The answers you give in the interview will be anonymized and kept confidential. The results will be used anonymously for research purposes in the context of the SIPATH project only.

Participation in this study is voluntary: You don't have to answer any question you don't want to and you can stop the interview at any time.

In order to evaluate your answers, it is important for us to record the interview.

☐ Yes, it is ok to record the interview.

With my signature, I confirm that I have understood the above information and voluntarily agree to participate in this survey.

Name of participant: ____

Signature ______________________________ Date ______________________________

If you have any questions concerning this study, please do not hesitate to contact us: Contact Data

Information about interview situation

Please write down the following information for each interview. They will be again used as the preamble of the transcript.

Interviewer:	
Code interviewee:	
Date & time of interview:	
Location of interview (*e.g. Kitchen of interviewee, Café,…*):	
Type of interview:	□ Face to face<br>□ Other: ______________
Duration of interview:	
Comments on interview situation (atmosphere, disturbances, observations):	
Sex of interviewee:	□ Female<br>□ Male<br>□ Other<br>□ No answer
Age of interviewee:<br>(take answer from 1.4)	

Part 1: Open questions about farmer's perception on change in agriculture*

A: Could you describe your life in agriculture?

- *During which time did you work in agriculture? (Starting year, Kolkhozes, transition time, >2000)?*
- *How did the profession of the farmer change between the time of taking over the farm and today?*

B: How satisfactory was the farming life [=your job on the farm] to you?

- *Did this vary during the time as an active farmer? Why did this vary? For interviewees working both in socialist and post-socialist times on a farm: Does it differ?*
- *What was satisfactory?*

C: What were (dis)stressful times for you when you were working in agriculture?

- *What caused the stress? / How did it impact you? / How did this change?*
- *Key words: Economic reasons, financial constraints, workload, insecurity regarding land tenure…*

D: How easy was it to make a living from agriculture? [= *How was the economic situation of the farm]*

- *When & why did this change over time??*
- *Was there a lot of income variability between single years? If yes, why?*

E: What were the main disturbances / shocks (*natural, political, social or economic*) that your farm experienced?

- *When and why? How did you react?*
- *Examples for shocks: Market fluctuations, new policies, collapse of political system, natural events such as floods, heat years or soil erosion, plant & animal epidemics, ...*

F: How did the people (farmers / villagers) within the community support each other?

- *How and why did this change?*
- *Support could include: Exchanging goods, services (helping out on the farm), information, etc.*
- *Did this exchange happen between neighboring farms or rather on a municipal or regional level?*

G: Did you feel like society valued farm work?

- *Did this change since you started working in agriculture? Why?*
- *Did this impact the way you farmed?*

H: How, when and why did the landscape change around your farm / in the municipality over the years?

- *Based on maps / aerial images, we observed following change: xy. Can you comment on this?*
- *Why and when did it change?*

J: How has the diversity of birds, butterflies / insects and wild flowers / herbs changed since you started working in agriculture?

*Questions in *italic* could be asked, if the farmer is not elaborating too much by him/herself.

Part 2: Questionnaire about changes on the farm(s)

1	General questions

1.1. What type of farm did you work on?

1.1.1. Farm type: ☐ Arable farm ☐ Mixed farm ☐ Dairy farm ☐ Pig farm ☐ (other)_______

1.1.2. Business type: ☐ family farm ☐ corporate farm ☐ (other)_______

1.2 Details in relation to farm

Family farm ***-> for interviews on post socialist time***

F 1.2.1 When did you start working on the farm and when did you take it over?

F 1.2.2 When did you (/ do you plan to) hand over the farm?

F 1.2.3 Did (/ do you) have a successor?

F 1.2.4 What is your relationship to the successor (family, external)?

Corporate farm (= Collective farms, agricultural companies) ***-> for interviews on socialist time***

C 1.2.1 When did you start working for agri. companies / collective farms? / When did you stop?

C 1.2.2 Did you switch the farm during this time? If yes, when?

C 1.2.3 What did you do before / after you worked in the company / collective farms?

C. 1.2.4 What were your positions / tasks?

C 1.2.5 When did you / do you plan to retire?

1.3 How did you become a farmer? (*Vocational education, vocational master, university degree, …*)

1.4 What is your age?

2	Farm characteristics

2.1 Farm size

2.1.1 How much farm land was managed when you started **and** when you stopped working there [**ha**]?

2.1.2 If relevant: When and why did the size of the farm land change?

2.2 Land use

2.2.1 List the land use [*crop land / pasture / permanent crop (e.g. orchard) / forest*] of the farm and its area [%] for when you started **and** when you stopped working on the farm?

2.2.2 If relevant: How and why did these shares change?

2.3 Land tenure

2.3.1 How much of the farmland was owned / leased (%) when you started **and** when you stopped working on the farm?

2.3.2 If relevant: When and why did this change?

2.3.3 Did the type of land tenure influence how the land was used?

2.4 Follow-up questions land tenure

2.4.1 Owned land: How did you come into the possession of the land?

2.4.2 Leased land: From whom did you lease (private person, estate, commons, the municipality, etc.)? When and why did this change?

3 Arable land

3.1 Crop rotation

3.1.1 Which crops (if possible with share of area in %) were cultivated in the crop rotation when you started **and** when you stopped working on the farm?

3.1.2 Why and when did this change?

3.2 Crop yield per area

3.2.1 How did yield per area of the main crop change?

3.2.2 If relevant: When and why did the crop yield per area of the main crop change?

4 Livestock

4.1 Animal number & type

4.1.1 List the type and number of livestock on the farm when you started **and** when you stopped working there? (*If known, please also note down the total in livestock units (LSU)*)

4.1.2 If relevant: Why and when did the type / number of animals change?

4.2 Animal feed

4.2.1 How many days did the animals spend on pastures when you started **and** when you stopped working on the farm?

4.2.2 What was the composition (e.g. grass, hay, silage, concentrates) of the animal feed when you started **and** when you stopped working on the farm?

4.2.3 How did the quantity of concentrates change over time?

4.2.4 Where did animal feed come from (*produced on farm / local / country / global*)?

4.2.5 If relevant: When and why did the fodder strategy change?

4.3 Animal productivity (= *i.e. Milk yield / lactation for milk cows, age of slaughtering weight for meat animals, no. of eggs / year for laying hens*)

4.3.1 How did the productivity of animals evolve during the time you worked on the farm?

4.3.2 If relevant: What has changed, when and why?

5 Grassland

5.1 Usage

5.1.1 Was the grassland cut or grazed? Did that change?

5.1.2 If relevant: When and why did the usage change?

5.2 Number of usages (= no. of cuts or grazing frequency)

5.2.1 What was the average number of uses when you started **and** when you stopped working on the farm?

5.2.2 If relevant: When and why did the frequency change?

6 Permanent crops

6.1 Type of permanent crops (e.g. orchards)

6.1.1 What permanent crops did you have when you started **and** when you stopped working on the farm?

6.1.2 If relevant: When and why did the type of permanent crops change?

6.2. Number (/ area) of trees (e.g. number of apple trees)

6.2.1 How many trees were there (/ how big was the area) when you started **and** when you stopped working on the farm?

6.2.2 If relevant: When and why did the number of trees / the size of the area change?

7 Inputs & Melioration

7.1 Fertilizer

7.1.1 When was mineral fertilizer used for the first time on the farm? [Only for Kolkhoz?

7.1.2 Based on your experience, did fertilizer use (organic and mineral) increase / decrease / stay constant during the years you worked on the farm?

7.1.3 How did the composition of fertilizer *(% of manure, mineral fertilizer, etc*.) change during the years you worked on the farm?

7.1.4 Why and when did the composition and amount of fertilizer change?

7.2 Additives (antibiotics, herbicides, fungicides, insecticides)

7.2.1 When were the first antibiotics used on your farm?

7.2.2 How did the use of antibiotics develop (*more / equal / less*)?

7.2.3 When were the first herbicides **and** fungicides **and** insecticides used on your farm?

7.2.4 How did the amount of herbicide **and** fungicide **and** insecticide use develop (*more / equal / less*)?

7.2.5 What were reasons to increase or decrease the amount of additives?

7.3 What farm measures did you implement to avoid soil degradation? How did this change?

7.4 Drainage

7.4.1 If relevant: How much [%] of the land was drained when you started **and** when you stopped working on the farm?

7.4.2 If relevant: When and why did the drained area change?

8	Innovation & Investments

8.1 Number of tractors

8.1.1 When was the first tractor purchased on the farm?

8.1.2 How many tractors were there on the farm, when you started **and** when you stopped working on the farm?

8.2 Mechanization

8.2.1 List other machines / technologies (*"which fundamentally changed the work processes on the farm*") that were purchased during the time you worked on the farm. ***Attention: concerns mechanization on the field & in the stable*** (*e.g. milking machines, feeding machines*).

8.2.2 When and why were new machines (*incl. tractors)* introduced?

8.3 Renting services / machines

8.3.1 What machines were rented / which work was done by contractors (= *machine + operator*) when you started **and** when you stopped working on the farm?

8.3.2 If relevant: When and why were more / less / other machines or contractors rented?

8.4 Infrastructure / Innovation

8.4.1 Did you / the farm management invest into other infrastructure or new technologies during the time you worked on the farm? If yes, what & when? (*e.g. stables, greenhouses, silos, roads*)

8.4.2 What was the motivation for these investments?

8.5 Background for decision-making

8.5.1 From where did you / the farm management get the information for new machines / technologies / inputs? Did this change over time? (*e.g. newspapers, agricultural machinery fairs, company representatives, training*)?

8.5.2 With whom (*i.e. family / friends / agricultural neighbors / comrades*) did you / the farm management discuss the purchase of new machines / the changing of practices? Did this change over time?

9 Working force

9.1 Year-round workers

9.1.1 How many people worked on the farm during the whole year when you started working **and** when you stopped on the farm? Why did it change?

9.1.2 If not mentioned already in 9.1.1: What was the role of family members (***partner/spouse,*** *parents, children*)? / How did that change?

9.2 Seasonal / harvest workers

9.2.1 How many seasonal / harvest workers were employed when you started **and** when you stopped working on the farm? Why did it change?

9.2.2 For which work and for which time period where seasonal / harvest works employed? How did that change?

9.2.3 Where did the seasonal / harvest workers come from (*e.g. municipality / country / abroad*)? If they were non-local, through which channels / networks are they hired?

9.3 Workload

9.3.1 Has the working time / day changed for you over the years? If so, when and why?

9.3.2 Were you able to take a vacation? If so, from when on, how did it change?

9.4 Off farm income

9.4.1 For family farms: Have you [or somebody else of the family] been also working off-farm when you started working **and** when you stopped on the farm? What percentage of your income was generated by off-farm employment?

9.4.2 If relevant: When and why did this change?

10 Farm strategies

10.1 Financial rewarding activities

10.1.1 What activities on the farm were financially most rewarding when you started **and** when you stopped working on the farm?

10.1.2 What % of farm revenue was generated by this product(s) when you started **and** when you stopped working on the farm?

10.1.3 If relevant: When and why did this change?

10.2 Labels / certification

10.2.1 What was the importance of labels / certification for the farm? How did this change?

10.2.2 If relevant: What was the cost (*e.g. monetary or organizational*) to get them?

10.2.3 What was the motivation (not) to get a label?

10.3 Sale market

10.3.1 Which sale markets were important for the main production(s) when you started **and** when you stopped working on the farm?

10.3.2 If relevant: When and why did the sales markets change? (*Where did you get information about alternative markets?*)

11 Policies & regional development

11.1 What were important regulations that the government implemented during your working years? Why were they important?

11.2 How did they influence the development of the farm?

11.3 If not mentioned: How did the importance of direct payments for the profitability of the farm change? How did direct payments / state support influence the orientation of the farm?

11.4 Changes in number of farms

11.4.1 Why did the amount of farms decline (*/ increase -> check with statistics before*)?

11.4.2 What happened to the land of the farmers that stopped farming? / Did this change over time?

11.4.3 What are the characteristics of the farms that still exist as opposed to those that have given up?

Finishing questions

- Is there anything you wish to add to our conversation today?
- If we still would have a follow-up question, may we contact you by phone?
- Would you be interested to be informed about the results of the study?
