# Supplementary material for: Why has farming in Europe changed? A farmers’ perspective on the development since the 1960s
Source: Reg Environ Change. 2023 Nov 11;23(4):156. doi: 10.1007/s10113-023-02150-y (PMC10640510; doi:10.1007/s10113-023-02150-y)
Supplement: Supplementary file 3 — Supplementary file3 (PDF 127 KB) [file 10113_2023_2150_MOESM3_ESM.pdf]

**Supplementary material:** Why has farming in Europe changed? A farmers' perspective on the development since the 1960s (Mohr et al.)

# Appendix III: Description of the identified subcodes by driving forces category

| **Driving forces categories** (adapted after Bürgi et al. 2004) | **Identified subcodes** | **Description** |
|---|---|---|
| cultural & personal | change in demography | Change in demography; e.g. increase in population due to suburbanization or decrease due to rural exodus. |
| cultural & personal | cooperation | Cooperation among farmers and/or within the village community made it possible to tackle agricultural tasks that would be too much for an individual. Cooperation also increased resilience, when e.g. the farming community câme to help when another farmer was injured. It is usually mentioned that cooperation decreased across time with some exceptions (especially in Hedmark, NO). |
| cultural & personal | farmer information exchange | Changes on the farm that depended on exchange with other farmers within informal networks. |
| cultural & personal | individual condition | Farm changes related to the interviewee's biography, e.g. changes to reduce workload to care for children or adaptation of special technology due to an accident. |
| cultural & personal | inheritance customs | Inheritance laws influenced farm size and/or farm manageability/farm price. |
| cultural & personal | intergenerational arrangements | Family matters: Communication between the two generations to (not) secure arrangements between the two generations. Usually measures are taken to facilitate a smooth transition of the farm to the next generation (e.g. joint ownership, early retirement, investment to hand over an already modernised farm). Sometimes, however, parents actively discouraged their children from following in their footsteps. |

| | | |
|---|---|---|
| cultural & personal | no interest to work in agriculture / no successor | Agriculture as an unattractive career for the farmer's children/family, often due to the economic, social or work-life balance disadvantages associated with running a farm. This has often resulted in the younger generation choosing other careers and eventually the older generation leaving the farm. Once it was known that the younger generation would not follow, the farm was usually not developed further. |
| cultural & personal | perceived good practice | Rationales for (no) change that were not attributed to a clear, reflected cause, but were rooted in what was culturally perceived as good practice. These included phrases such as "this was the way to do it", “according to our needs”, “we had to”, etc.. |
| cultural & personal | personal preferences / attitude | Personal motivation, beliefs, values, e.g. farmers renting out their land after retirement rather than selling it to keep the connection, or doing something to protect nature because they feel it is personally important. |
| economic | contract farming & similar schemes | Schemes that provided secure outlets/prices for the farmer (e.g. supermarket chains). But they can also lead to farmers being caught up in a web of power. |
| economic | demand in [farm] services | Opportunities for the farmer to earn extra income through additional activities on the farm, such as renting rooms to students or tourists, or doing contract work for other farmers. |
| economic | farm strategic planning [grow] | Expansion of the farm (e.g. size [ha], number of animals) in order to remain competitive in the face of structural change/general economic pressure, but also own will/initiative to keep up with this trend. |
| economic | farm strategic planning [niche] | Farmers have chosen to move into a niche that has allowed them to increase their economic returns without increasing the physical size of the farm or pursuing economies of scale in production. |
| economic | farm strategic planning [optimisation & self-reliance] | Farm management decisions to optimise work flow and farm budget to remain competitive in the face of structural change/general economic pressures. For example<br>-New machinery to save time or reduce the number of workers<br>-Using alternatives to investing in own equipment |

| | | |
|---|---|---|
| | | (contractor service), because for certain jobs it is cheaper to rent services/machines than to invest in them.<br>-owning machinery to be able to harvest when the weather is most favourable. |
| economic | farm strategic planning [productivity increase] | Farm management decisions to increase productivity (= increase in yield/unit) without changing the size of the farm in order to remain competitive in the face of structural change/general economic pressure, e.g. decision to switch to a different breed of dairy cow to get more milk per cow. |
| economic | improve /maintain quality of soil/land | Invest to improve or maintain the quality of the soil/land to ensure higher/stable yields in the future. For example, maintaining terraces or draining and levelling land. |
| economic | land as investment | Agricultural land is bought as an investment by non-agricultural actors, increasing competition for land from farmers. |
| economic | market [change in price] | Changes in commodity prices lead to adjustments on the farm, e.g. a prolonged decline in the commodity price leads to economic hardship, which eventually leads to a change in farming strategy or abandonment of the farm. |
| economic | market [conditions / access] | Market opportunities/access to certain markets or lack thereof influenced farm management/investment strategies. For example, access to stable, good market prices meant that olive oil farmers invested heavily in more olive trees; or after the collapse of the socialist system, access to markets to sell agricultural produce was difficult, leading to fragile economic viability. |
| economic | market [gaining more agency] | In order to gain more bargaining power and thus better economic viability, certain measures have been taken, such as setting up a cooperative to better market agricultural products or investing in warehouses to wait for crop prices to rise. |
| economic | market [increase in / high operating costs] | An increase or continuation of high operating costs (e.g. feed price, fertiliser price) triggers adjustments in farm management. |
| economic | market [price fluctuation] | Continued price fluctuations have influenced decisions on which crops to grow or lead to diversification of farm strategies. |

| | | |
|---|---|---|
| economic | market-based labelling instruments | The possibility of market-based labelling instruments (e.g. organic, protected designation of origin) drove the farm towards developments that differ from typical farm consolidation strategies. |
| economic | off-farm employment opportunities | Employment opportunities outside agriculture. This could result in fewer people working in agriculture or in farmers / farm families being able to earn a second income. In the latter case, off-farm employment was often cited as being less time-consuming and a more profitable way of making ends meet. |
| economic | risk aversion | Certain measures (e.g. insurance) have been taken to reduce the risk of crop failure or disease. |
| economic | availability of (skilled) labourers | Certain farming strategies have been enabled or hindered by the availability or scarcity of (skilled) workforce. |
| economic | socialist system [increase economic return / yields] | Increase in profit, driven by the collectivised farm/kolkhoze's own motivation, which exceeds the target set by the planned economy. The profit can be used to invest more in the collectivised farm/kolkhoze, in the common life of the members (e.g. vacation home) or in the community. |
| economic | socialist system [private side business] | Opportunities for sideline farming complemented the land use/agricultural production of the collectivised farm/kolkhoze and provided (mostly) members with a welcome second income. |
| economic | trust / bonds to market outlets | Trust or bonds to market outlets lead to decisions that were not driven by the best economic return, but by a sense of stability or loyalty. |
| institutional | advisory, training and supporting institutions | Institutions such as the state, trade unions, cooperatives or private companies provide training and advice that has influenced farm development (e.g. the amount of pesticides sprayed); during the GDR, cooperation with science and related large-scale field experiments (e.g. the introduction of driving lanes; sewage irrigation system; use of antibiotics) also influenced farm development. |
| institutional | availability of land / prevalent tenure schemes | Availability of land / prevalent land tenure systems allow or inhibit certain developments. |

| | | |
|---|---|---|
| institutional | change in system boundaries | Changes in system boundaries affected the viability of farms/triggered farm changes. For example, the end of the communist system meant that farms had to adapt to the capitalist system (e.g. reduce the number of people working on the farm). Or changes in foreign policy/trade barriers allowed people to move to other places and/or other goods to enter the country, affecting e.g. commodity prices. |
| institutional | collectivisation [initial] | Initial collectivisation, which resulted in collectivised farms/kolkhozes that were still relatively small in size and with varying degrees of collectivisation/management hierarchy. |
| institutional | collectivisation [re-organisation] | The reorganisation of the kolkhozes led to far-reaching changes, such as changes in size, the separation of animal and plant production, or the creation of other institutions such as the ACZ (Agrochemical Centre) in the GDR. During this period, agriculture was intensified thanks to new technologies. |
| institutional | formal education & system knowledge | On-farm change decisions were influenced by formal education and system knowledge. |
| institutional | further financial / material incentives | Smaller/less frequent government contributions to encourage farmers to take certain actions: e.g. encouraging or supporting the organisation of fertiliser/additive use through low prices, usually leading to increased fertiliser use; one-off financial incentive to cut field trees. |
| institutional | land consolidation | Land consolidation led to a redistribution of land and often an increase in the size of fields. Land consolidation often made more land available for purchase by existing farmers. Land consolidation was also organised by a private group of farmers. |
| institutional | land reform | The land reform led to a redistribution of land and thus changed the shape of farms and actors in agriculture. In this analysis, the socialist land reform has mainly been mentioned. |
| institutional | planned economy [availability of resources] | Top-down decisions determined which and how much resources (e.g. building materials, fodder, fertiliser, machinery) were available to farms. This was sometimes perceived as a constraint (especially in terms of available fodder for dairy cows), but it also stimulated creativity in obtaining resources in other ways. |

| | | |
|---|---|---|
| institutional | planned economy [farm management] | Top-down decisions determined the direction of the farm and what/how much should be produced. |
| institutional | planned economy [labor organization] | Top-down work organisation, resulting in, for example, little influence on the choice of wages or the number of workers employed, predetermined careers, assignment of people with disabilities/unemployment to farms without consultation with farm leaders. |
| institutional | policy uncertainty | A lack of reliability and transparency made it difficult to calculate long-term investments for the farm and sometimes led to misinvestments that affected the viability of the farm; it also negatively influenced the farmers' view of government schemes. |
| institutional | post-socialist transition | Transitional measures taken by the state to ease the turbulent times of transition to a liberal market economy; redistribution of land after the collapse of the socialist regime, such as the transformation of collectivised farms/kolkhozes into cooperatives/enterprises, the reclaiming of farmers' land, or the schemes that leased/sold formerly state-owned land (e.g. the so-called "Treuhand" in the GDR). |
| institutional | quality control of commodities & sanitary controls | Quality control of raw materials and health inspections raise awareness of animal diseases and can lead to adaptation of farm strategies (e.g. change of cow breed after mass culling as a result of a detected disease). |
| institutional | quota | Government or company imposed quotas, or their removal, affected the number of commodities that could be produced by the farm, and thus affected farm strategy. |
| institutional | restrictions / standards for farming operations [ecology] | Farm management has been constrained by rules/standards designed to promote more ecological farming (e.g. reduced use of fertilisers). Failure to comply with these rules was often sanctioned by a reduction in direct payments. |
| institutional | rural development policy [case-study specific] | Local rural development policies (natural, social, economic) that affect farmers as rural actors. E.g. development of residential areas at the expense of agricultural land. |
| institutional | social regulations | Social regulation, such as compulsory health insurance for farmers, has resulted in benefits and/or increased operating costs for farms. |

| | | |
|---|---|---|
| institutional | state support for credits / machinery | Easy access for farmers to loans or subsidies for certain machinery to support farmers, which influenced e.g. technology adoption, ability to farm larger areas. This subcode also includes subsidy/loan schemes for young farmers. |
| institutional | subsidies | Subsidies as an important influence on farm strategy and land use intensity. Both 'early' subsidies for selected commodities/quantity based and area subsidies/direct payments. The shift towards direct payments was often pronounced, e.g. in France, where some farmers reported cutting down parts of the bocages (hedges) to increase the total area. |
| institutional | times of turnover and crisis | In times of turnover and disasters, new regulations, actors, etc. appeared temporarily, making business as usual impossible and affecting the viability of the farm. E.g. dubious actors deceiving farmers after the collapse of the socialist system. |
| institutional | voluntary ecological schemes | Voluntary ecological schemes have given farmers the opportunity to earn money / adapt strategy while changing their practices towards more environmentally friendly farming. |
| natural | frequency & intensity of weather events | Single but severe weather events that have influenced farming decisions. Also frequency of e.g. droughts in recent years and resulting adaptations. |
| natural | local climate & water availability | Constant location factor influencing which farm strategies were possible/viable. |
| natural | pests & pathogens | Animals (e.g. mice, wild boar), insects and diseases that adversely affected the agricultural yield of (perennial) crops. This was often linked to the resulting economic difficulties, e.g. when the disease resulted in mass culling or a large proportion of the yield was lost in mouse years. |
| natural | soil conditions | Soil conditions as a location factor leading to a certain strategic development of a farm. Often related to unfavourable soil conditions that limited the possible uses. |
| natural | spatial configuration | General regional / on-farm spatial organisation that enabled / prohibited certain trajectories of change, e.g. if the grasslands were far from the farm, it is not possible to let the cattle graze. |

| | | |
|---|---|---|
| natural | topography | Topography as a location factor that enabled or limited farm strategies, especially with regard to mechanisation. For example, in the case of olive growers in Lesvos, the hilly terrain did not allow mechanisation of olive cultivation and therefore intensification. |
| technological | additives | The introduction and development of pesticides and antibiotics reduced labour (e.g. weeding), increased resilience to pests in the short term and were generally associated with input intensification in agriculture. |
| technological | breeds | The introduction and improvement of new breeds, selective breeding, insemination, sexed semen, etc. made it possible to increase the productivity of livestock. |
| technological | feed | Increased knowledge of how to feed new, intensive breeds and the use/availability of new types of feed (concentrates) increased livestock productivity. |
| technological | fertiliser | Introduction and further development of mineral fertilisers increased production; new application strategies for animal manure made its use on larger areas possible/viable. |
| technological | information management | New opportunities for efficient farm management thanks to the digitalisation of operations (GPS, digital control of machinery) reduced working time and increased efficiency. |
| technological | introduction / renewal of irrigation system | Introduction or renewal of irrigation as a game changer for agriculture in dry areas, or as a temporary installation in temperate areas in dry years to enable a good harvest. |
| technological | machines [field usage] | New types and refinements of mobile machinery used in the fields that reduced labour and allowed farmers to farm larger areas. E.g. tractors, precision seed drills, pesticide sprayers, loader wagons. |
| technological | on-farm infrastructure | Stationary infrastructure (e.g. new free stall barn, introduction of milking machines, silo, packaging machine, olive netting, fencing) that e.g. facilitated work and made it more efficient / less dependent on external influences. |
| technological | regional infrastructure | Creation of new regional infrastructure that has changed farming opportunities, such as access to transport infrastructure/roads or water/electricity networks. |
| technological | seeds | Introduction and/or development of seed varieties (e.g. leading to higher yields or more resistant crops) |
