# Supplementary material for: Why has farming in Europe changed? A farmers’ perspective on the development since the 1960s
Source: Reg Environ Change. 2023 Nov 11;23(4):156. doi: 10.1007/s10113-023-02150-y (PMC10640510; doi:10.1007/s10113-023-02150-y)
Supplement: Supplementary file 4 — Supplementary file4 (PDF 256 KB) [file 10113_2023_2150_MOESM4_ESM.pdf]

Supplementary material: Why has farming in Europe changed? A farmers' perspective on the development since the 1960s (Mohr et al.)

Appendix IV: Overview coding

In the following flow chart, an overview over the different coding steps is given:

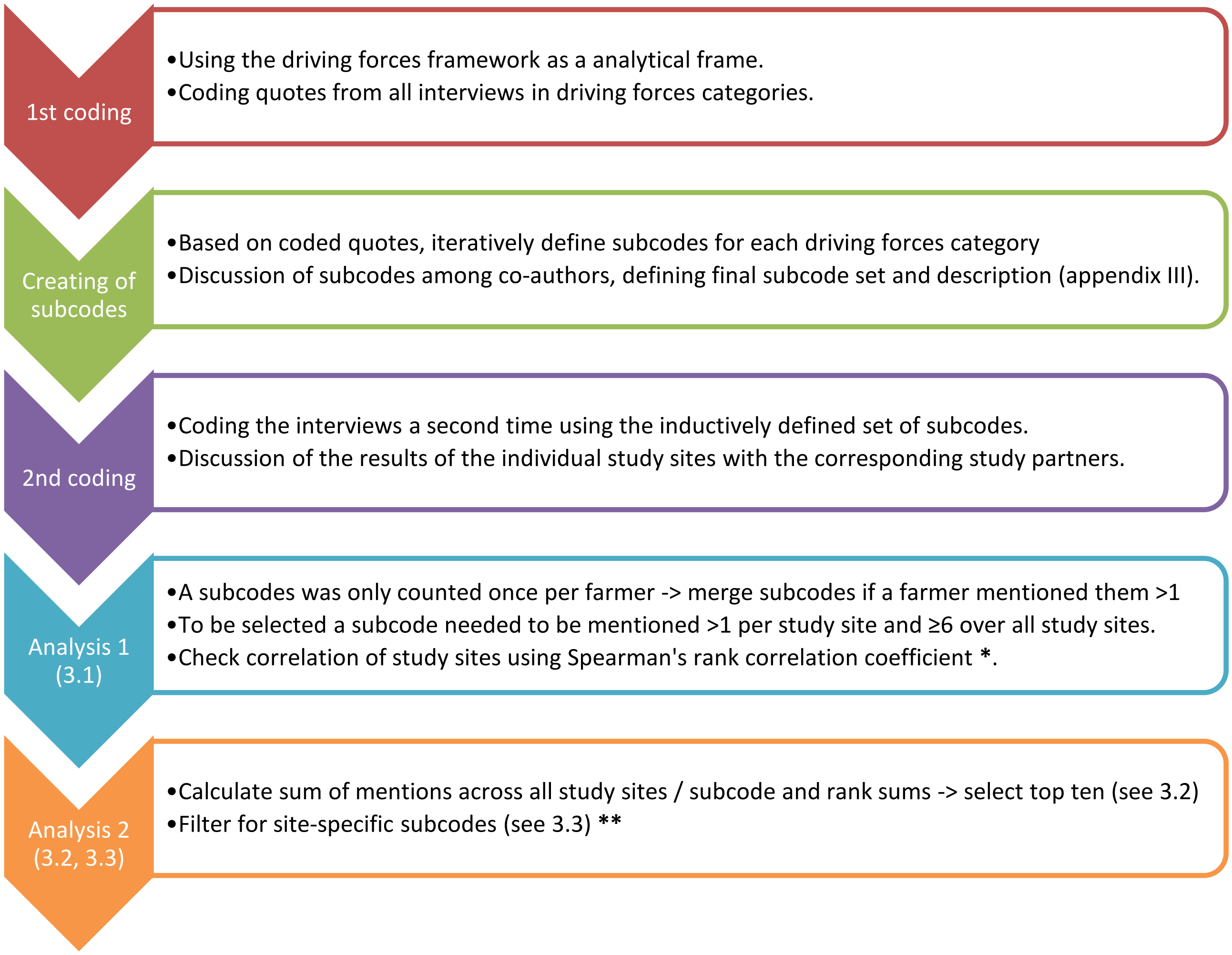

Figure 1: Description of the coding steps.

Regional environmental change; franziska.mohr@wsl.ch

* Spearman's rank correlation coefficient shows the following correlation between the study sites:

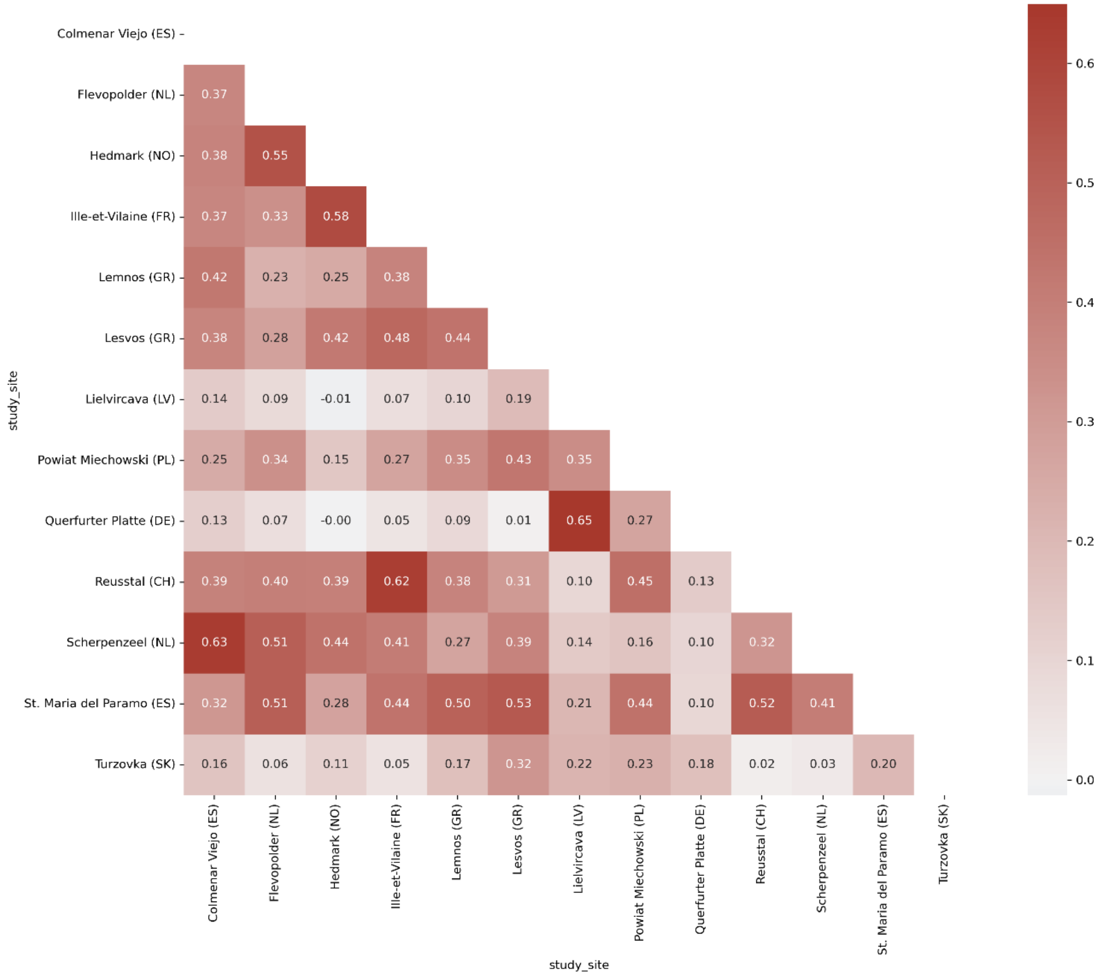

Figure 2 Confusion matrix showing the correlation between the study sites (Spearman's rank correlation coefficient).

** To filter for site-specific subcodes/driving forces, we calculated the following for each study site (the calculations were done using Python/Pandas):

1) All subcodes were ranked based on overall sum of mentions across all study sites (i.e., of which the ten highest ranking subcodes are shown in 3.2, Figure 5).
2) The subcodes were ranked based on mentions in individual study site.
3) The difference between 1) and 2) were calculated and the difference ranked.
4) The site-specific subcodes were selected for the individual study site all the following 3 criteria were met:
   a) The subcode is among the top 10 ranked subcodes for the individual study site (see step 2).
   b) The subcode is among the top 10 ranked differences (see step 3).
   c) Control condition: The subcode is among the overall top 10 ranked differences (see step 1).
5) The resulting selection of subcodes was then visualized in a heat map (see subchapter 3.3, Figure 6).
